# Exploring the Validity of Adolescent Responses to a Measure of Psychological Flexibility and Inflexibility

**DOI:** 10.3390/bs15020197

**Published:** 2025-02-12

**Authors:** Caleb D. Farley, Tyler L. Renshaw

**Affiliations:** Department of Psychology, Utah State University, 2810 Old Main Hill, Logan, UT 84322, USA; caleb.farley@usu.edu

**Keywords:** psychological flexibility, psychological inflexibility, adolescents, measurement, validity, mental health, screening

## Abstract

Validating measures of psychological flexibility (PF) and psychological inflexibility (PI) has occurred in multiple adult samples, but little research has validated PF and PI measures with adolescents. This manuscript describes two studies exploring the validity of responses to the Multidimensional Psychological Flexibility Inventory (MPFI) with two samples of adolescents. The first study used exploratory factor analyses on responses to the MPFI with a sample of 16–17-year-olds (*N* = 249). The results yielded a reduced and simplified measurement model that consisted of two general factors: one for PF and the other for PI. These exploratory findings were further investigated with confirmatory factor analyses in the second study, with a larger sample of 14–17-year-olds (*N* = 503). The results from the second study generally confirmed the factor model from the first study. Findings from both studies showed that scores derived from the reduced MPFI measurement model evidenced convergent and divergent validity with a variety of mental health criterion measures. Moreover, findings from the second study showed that PF and PI scores had differential predictive power on different concurrent mental health outcomes. This discussion highlights the implications of measuring PF and PI in adolescents, considers limitations of the present studies, and recommends next steps for research.

## 1. Exploring the Validity of Adolescent Responses to a Measure of Psychological Flexibility and Inflexibility

Researchers estimate that a growing number of youth in the United States are struggling with their behavioral health, most notably an uptake in suicide risk (Twenge et al., 2019). Schools, primary medical sites, and outpatient agencies have influence over the care and behaviors of most youth, including those from low-income families and other groups at risk (Ali et al., 2019; Friman, 2010). Task forces are increasing the utilization of these public systems to support the wellbeing of families (Ali et al., 2019; Friman, 2010) through, in part, brief, targeted screeners that place and track youth in appropriate, preventative services (Kilgus & von der Embse, 2019). Effective assessments discriminate the scope and severity of risk factors and point families towards necessary levels of support (Kilgus & von der Embse, 2019). Such screening includes identifying universally maladaptive or adaptive transdiagnostic behaviors that predict multiple psychological disorders (Follette et al., 2000; Harvey, 2014). This type of screening should be capable of administration across multiple populations to assess the frequency of a behavior that is variable, amenable to change, and predictive of diverse psychopathologies (Coyne et al., 2011). Questions thus arise surrounding certain skills or behaviors that are most useful to screen for to inform more intensive assessments or targeted interventions.

An inability to maintain functional behaviors across contexts, manifested as behavioral rigidity from receiving desired consequences, is related to a host of psychopathologies (Follette et al., 2000). Pivoting one’s behavior functionally may be a useful, identifiable trait that differentiates those at risk for behavioral problems (Nolen-Hoeksema & Watkins, 2011). A measure of this trait would point youth to interventions effective at increasing the flexibility they present a deficiency in (Biglan & Hayes, 1996). Psychological flexibility (PF) and the inverse, psychological inflexibility (PI), are dual-nature traits that may represent this ability (Renshaw, 2017).

### 1.1. Theory of Psychological Flexibility and Inflexibility

Psychological flexibility (PF) is “the ability to contact the present moment more fully as a conscious human being, and to change or persist in behavior when doing so serves valued ends” (S. C. Hayes et al., 2006, p. 7). PF is based upon relational frame theory, which suggests that individuals with developed cognition can behave differently from determined rules and beliefs about themselves (Ong & Eustis, 2023). People with greater PF can respond in different and adaptive ways toward reinforcements and punishments, regardless of what their thoughts and feelings present (Ong & Eustis, 2023). In contrast, psychological inflexibility (PI) is rigidly responding in accordance with the thoughts or feelings that are present, which may differ from outward reinforcements or punishments they experience (S. C. Hayes et al., 2006).

This skill of PF includes six processes, each contributing to a general construct: present-moment awareness, cognitive defusion, acceptance, self-as-context, values, and committed action (S. C. Hayes et al., 2006). Being psychologically flexible is showing competency across all processes (Ong & Eustis, 2023). PI, on the other hand, involves six inverse processes: lack of present-moment awareness, cognitive fusion, experiential avoidance, self-as-content, lack of contact with values, and inaction (S. C. Hayes et al., 2006). Being psychologically inflexible is showing a frequency of all inverse processes (Ong & Eustis, 2023). PF and PI have been claimed to represent dual-construct behaviors, such that one who is more psychologically inflexible is likewise less psychologically flexible (Ong & Eustis, 2023).

These processes that contribute to PF and PI (e.g., acceptance, defusion, lack of values, etc.) are shown to predict various reported levels of psychopathology and wellbeing (Rolffs et al., 2018). For instance, greater acceptance is associated with a greater ability to navigate chronic pain, higher levels of wellbeing, and increased engagement in adaptive behaviors (Hershenberg et al., 2017). Engagement with defusion exercises is related to increases in pain tolerance and decreases in emotional distress (e.g., Carrasquillo & Zettle, 2014). Present-moment awareness is associated with reduced stress, improved attention, and increased emotion regulation (Brown & Ryan, 2003), and increases in self-as-context have relatedly predicted improvements in functional impairment at work, depression, and pain endurance (Godbee & Kangas, 2020). Understanding values and consistently behaving in accordance with them have been associated with increased wellbeing and physical health, as well as decreased clinical pathology (Stapleton et al., 2020). Finally, clients reporting higher levels of committed action also reported sleep improvements, decreased pain, greater wellbeing at work, greater social adjustment, and decreased depression and fatigue (Daly-Eichenhardt et al., 2016).

Given this research, scholars suggest that these processes should be taught with brief, targeted interventions to large groups, which may prove useful for increasing the wellbeing of individuals broadly (Levin et al., 2014; Levin et al., 2016). Early theory suggests that this may also be true for adolescent populations (Renshaw et al., 2023). For instance, a recent longitudinal study showed that higher levels of reported acceptance and present-moment awareness were predictive of greater prosocial behaviors and wellbeing in 10th-grade adolescents over one year of school (Ciarrochi et al., 2011). In another study, low emotional identification ability predicted adolescents’ increased levels of positive affect, with the authors highlighting the importance of present-moment awareness and self-as-process fostering desirable affective experiences (Ciarrochi et al., 2008). Finally, a study showed that youth writing about their most important values improved grades and decreased reported levels of stress (Cohen & Sherman, 2014). Data also showed that interventions targeting PF and PI in youth were related to increased reports of wellbeing and reduced levels of internalizing problems (Harris & Samuel, 2020). Experiential avoidance and fusion, measured by the Avoidance and Fusion Scale for Youth (AFQY; Greco et al., 2008), is related to decreases in different adolescent clinical psychopathologies (Livheim et al., 2020). The AFQY relatedly demonstrated clinical utility in effectively screening for anxiety and depression disorders, transdiagnostically, in a school-based population (Renshaw, 2017). Since processes of PF and PI show promise in predicting a broad range of mental health outcomes, more attention is being given to these combined processes, measured as PF and PI broadly. However, research regarding the measurement of these constructs in adolescents has been minimal to date.

### 1.2. Measuring Psychological Flexibility and Inflexibility

PF and PI are measured at different scopes with varying levels of definition and detail, mostly in adults older than 18 and barely with youth. Used in adults at the most detailed and comprehensive level is Rolffs et al.’s (2018) Multidimensional Psychological Flexibility Inventory (MPFI), comprising 12 distinct factors, each represented by six items, together representing the six positive and six negative processes (Ong et al., 2024). Each of the two total scores contributes to correlated, higher-order factors representing PF and PI. The structure designed by Rolffs et al. (2018) was validated again with adults in both Seidler et al. (2020) and Thomas et al. (2022). That said, upon probing the convergent validity of these processes in older adolescents, problematic relationships were shown, such that the acceptance factor correlated with processes representing inflexibility, and the experiential avoidance factor correlated with processes representing flexibility (Barr, 2022).

At a mid-tier level, a very recent study examining adult responses to the MPFI showed that the six PF processes did not separate into six distinct components (Christodoulou et al., 2023). Values and committed action combined as one process and self-as-context and present-moment awareness as another; however, neither acceptance nor defusion item responses contributed as central components to the overarching structure (Christodoulou et al., 2023). Reflecting on these findings, Christodoulou et al. recommended a sounder three-factor structure of PF in which acceptance and defusion are combined into an “open” factor, present-moment awareness and self-as-context are combined into an “aware” factor, and values and committed action are combined into an “engaged” factor. This aligns with theory suggesting that PF might be more effectively measured via a three-factor structure representing openness, awareness, and engagement, and PI might be likewise measured as not open, not aware, and not engaged (S. C. Hayes et al., 2011). Related to this recommendation exists broader measurement tools like the Comprehensive Assessment of Acceptance and Commitment Therapy (compACT; Francis et al., 2016), which measures PF in adults. In this measure, present-moment awareness- and self-as-context-related items represent behavioral awareness, acceptance- and defusion-related items represent openness to experience, and values- and committed action-related items represent valued action (Francis et al., 2016). To date, neither the compACT nor other condensed structures have been explored or validated in adolescents.

At a broader level of assessment exists partial measures of PI, operationalized as single, unified structures sans processes or subscales. Greco et al.’s (2008) Avoidance and Fusion Questionnaire for Youth (AFQY) is adequately validated for youth (Petersen et al., 2023). The AFQY has shown transdiagnostic utility for screening youth with anxiety and depressive disorders in school-based populations, predicting youth wellbeing, and correlating with other measures of experiential avoidance (Renshaw, 2017). The AFQY demonstrates sound structural proprieties and maintains theoretically consistent relationships with appropriate criterion variables (Greco et al., 2008). However, although successful, this measure and the measure for adults that it was based on, the Acceptance and Action Questionnaire–II, have been critiqued as inadequate representations of the PF and PI dual constructs, given their narrow, specific foci. (McLoughlin & Roche, 2023). For example, items representing contact with values are not included in either measure, and neither represent PF, since all items are negatively phrased (McLoughlin & Roche, 2023; Ong et al., 2024). These different approaches demonstrate significant variation across self-report measures of PF and PI in adults and show minimal validation efforts have been conducted in adolescents. Research is thus warranted to intentionally validate PF and PI measures in younger populations.

### 1.3. Purpose of Present Study

Measuring PF and PI in adolescents may aid in identifying, treating, and monitoring their behavioral health. Forms exist that measure certain processes partially related to PF and PI (e.g., mindfulness, experiential avoidance, values, etc.). However, no validation research has shown how all combined processes of PF and PI contribute to comprehensive factors in adolescents (McLoughlin & Roche, 2023; Petersen et al., 2023). While some measures exist for adults, targeted validation in younger populations is important because adolescents experience different biological, developmental, social, and emotional contexts than adults (Steinberg, 2005). Adolescents experience greater amounts of emotions, decreased sense of identity, and increased need for adaptivity compared with adults (L. L. Hayes & Ciarrochi, 2015). They also show decreased ability in abstract thinking, self-reflection, and long-term evaluations (Piaget, 1971). Rating forms require cognitive reflections of one’s own experiences, so validation efforts specific to adolescents must involve simplification and developmental adaptation. Measures validated with adults may be unreliable at representing experiences in adolescence given the limited self-reporting ability they likely possess.

This paper thus explores the structural, convergent, divergent, and predictive validity of PF and PI in adolescents through examining adolescent responses to the most comprehensive and detailed measure of PF and PI for adults to date, the MPFI. This was accomplished through conducting two studies: an exploratory pilot investigation of responses from a smaller, pre-existing sample of adolescents across the United States and a replication confirmatory validation on a similar but larger sample. Factor analysis was used to examine a structural model of PF and PI in adolescents through highlighting relationships among a large set of observed variables that predicted certain latent factors (Flora & Flake, 2017). Exploratory factor analysis (EFA) first used in Study 1 lacked regard for a hypothesis about an optimal number of factors (Flora & Flake, 2017), which was necessary to explore the pre-existing dataset since little work has yet explored if all processes of PF and PI actually contribute to general factors (Petersen et al., 2023). Since past research shows all PF and PI factors contribute to PF and PI constructs in adults, then the initial EFA in Study 1 should show that the same items should contribute wholistically to PF and PI factors in adolescents. Following EFA, bivariate correlations were analyzed in Study 1 between the identified latent structure and related variables to evaluate convergent and discriminant validity.

The research questions guiding Study 1 were as follows:

1. Based on theoretical considerations and factor loadings from the EFA with responses to the MPFI, will the PF and PI models change or reduce to alternative structures (different from the original 12-factor MPFI model) that adequately maintain all latent variables?

2. Do scale scores derived from factors identified via EFA demonstrate theory-consistent relationships (convergent and divergent) with related measures, including experiential avoidance, internalizing problems, self-compassion, and wellbeing?

Based on findings from Study 1, Study 2 used an argument-based approach for measurement validation (Kane, 1992). In this approach, research should identify claims inherent in a proposed interpretation of a measure and then critically evaluate these claims using responses from the population of interest (Kane, 1992). Theory suggests that processes of PF (e.g., indicators of acceptance, present-moment awareness, values, etc.) contribute to a broader PF construct, and processes of PI (e.g., indicators of experiential avoidance, fusion, self-as-content, etc.) contribute to a broader PI construct. Theory claims that PF and PI should significantly and negatively correlate since one would assume high levels of PF should indicate low levels of PI (Ong & Eustis, 2023). PF and PI should relate and be predictive of relevant youth clinical processes in a theoretical manner. PI should thus correlate with and predict indicators of psychopathology, and PF should correlate with and predict indicators of wellbeing (Petersen et al., 2023). PF and PI constructs should also correlate and predict inverse processes to a significant extent, such that PF negatively correlates with psychopathology and PI negatively correlates with wellbeing.

Study 2 tested these claims through examining adolescent responses to multiple rating forms from a large, nationwide sample of individuals aged 14–17 years old. The identified factor model of PF and PI from Study 1 was examined using CFA on sample responses to the MPFI. Descriptive statistics of the observed scores from a simplified scale found from Study 1 were then calculated, entitled as the Multidimensional Psychological Flexibility Inventory for Youth (MPFI-Y). Bivariate correlations of the MPFI-Y were examined with related PF and PI process and desired mental health outcomes. Similar bivariate correlations were compared with the original observed PF and PI scores on the traditional 12-factor MPFI model. Finally, multivariate and binomial regression models were utilized to examine the value added of the reduced MPFI-Y scores on predicting clinically meaningful variables in adolescents, including internalized psychopathology, wellbeing, and suicide risk. 

The research questions guiding Study 2 were as follows:

1. Does CFA of the MPFI-Y’s factor structure yield adequate structural properties for measuring PF and PI?

2. Do observed scores representing PF and PI demonstrate theory-consistent relationships (convergent and divergent) with psychopathology and wellbeing variables? Relatedly, are these relationships comparable to or better than relationships observed with scores from the traditional MPFI 12-factor model?

3. To what extent do observed scores of PF and PI from the MPFI-Y model have incremental value in predicting adolescent reports of wellbeing and psychopathology?

## 2. Study 1

### 2.1. Materials and Methods

#### 2.1.1. Participants

Participants for this study came from a pre-existing dataset obtained by the original survey administration in a thesis study conducted by Barr (2022). The open dataset from Barr’s (2022) study was shared with the authors of the present study for the purpose of secondary data analysis. Collection of Barr’s (2022) dataset was approved by a university-centered institutional review board and obtained in partnership with Qualtrics survey panels, which is a market research panel that identifies a group of people to respond to surveys. They are typically chosen from a pre-arranged pool of respondents who have agreed to be contacted by market research in order to respond to the surveys. Data collection gathered a sample of adolescents (N = 249) aged 16–17 years that matched appropriate race and ethnicity ratios in the United States. A total of 175 participants identified as White or Caucasian (70.3%), 36 identified as Black or African American (14.5%), 36 identified as Hispanic, Latinx, or of Spanish origin (14.5%), 9 identified as American Indian or Alaska Native (3.6%), and less than 3% identified as Asian, Asian American, Middle Eastern, North African, Native Hawaiian, Pacific Islander, or other races. A total of 138 participants identified as Male/Man (55.4%), 107 as Female/Women (43%), and 4 as Transgender, Other, or “prefer not to answer” (1.6%). A total of 162 reported being 16 years old (65.1%), and 87 reported being 17 years old (34.9%). The readability of all measures administered was assessed as appropriate for youth 16 to 17 years of age.

#### 2.1.2. Data Collection

Participants received informed consent from a legal guardian and likewise offered assent before participating. Youth then responded to nine self-report forms, beginning with the MPFI and then others measuring experiential avoidance, anxiety and depression, wellbeing, and emotional coping.

#### 2.1.3. Measures

*Multidimensional Psychological Flexibility Inventory*. Rolffs et al.’s (2018) MPFI is a 60-item self-report measure that assesses all processes representing both PF and PI. The measure maintains a 12-factor structure with six representing PF (values, committed action, acceptance, defusion, present-moment awareness, and self-as-context) and six representing PI (lack of contact with values, inaction, experiential avoidance, fusion, lack of present-moment awareness, and self-as-content). Items are assigned point values on a scale of 1 to 6, with 1 = “Never True” and 6 = “Always True”, with higher scores indicating higher levels of each process. These observed scores can be averaged across processes/subscales as index scores of PF and PI. Items reflecting flexibility include: “I was able to let negative feelings come and go without getting caught up in them” and “Even when times got tough, I was still able to take steps toward what I value in life”. Items reflecting inflexibility include: “Negative thoughts and feelings tended to stick with me for a long time” and “Negative thoughts and feelings easily stalled out my plans”.

*Acceptance and Action Questionnaire–II.* Bond et al.’s (2011) AAQ-II is a representation of PI in adults as a 7-item self-report assessment using a 7-point Likert-type scale ranging from “1 = never true” to “7 = always true”, with higher scores indicating higher levels of PI. Items include “My painful memories prevent me from having a fulfilled life” and “Worries get in the way of my success”. Meyer et al. (2013) showed the measure to have good internal consistency and good test–retest reliability in adults. While initially validated for adults, the AAQ-II showed adequate convergent and divergent validity with other measures of youth inflexibility when administered to a sample of older adolescents (Barr, 2022).

*Avoidance and Fusion Questionnaire for Youth–17*. Like the AAQ-II, Greco et al.’s (2008) AFQY measures PI but is adapted for youth and adolescent populations. The measure utilizes a 5-point Likert-type scale ranging from “0 = Not true at all” to “4 = Very true”, with higher scores indicating increased levels of the construct. The measure has a long form with 17 items and a short form with 8 items, both of which have been confirmed as valid measures of the construct at hand (Greco et al., 2008). The current study utilized the 17-item version, and examples of items include the following: “I am afraid of my feelings” and “I can’t be a good friend when I’m upset”. Livheim et al. (2016) further showed the measure to have good internal consistency reliability in accordance with the youth populations it was designed to assess.

*Revised Child Anxiety and Depression Scale—25*. Ebesutani et al.’s (2012) original RCADS was designed to screen for the severity of depression and anxiety symptoms through 47 self-report items, and the RCADS-25 is a shortened version of that scale. The measure utilizes a 4-point response scale ranging from “0 = Never” to “3 = Always”. A global composite representing general internalizing symptoms was calculated for the present study. Higher summed scores are interpreted to represent greater amounts of internalizing problems, and the measure has been validated in multiple youth populations (Ebesutani et al., 2012; Piqueras et al., 2017).

*Subjective Happiness Scale*. Lyubomirsky and Lepper’s (1999) SHS is a brief scale of subjective happiness. The measure utilizes four items, with two focusing on general subjective happiness and the others on perceptions of one’s happiness in comparison to peers. Items are ranked along a scale ranging from 1–7, with higher scores indicating higher levels of happiness. Research shows this measure maintains good internal consistency and reliability in youth, and further evidence suggests consistent concurrent validity when vetted against other measures of mental health and wellbeing (Lyubomirsky & Lepper, 1999; Renshaw, 2018).

*Satisfaction with Life Scale*. Another measure of wellbeing, Diener et al.’s (1985) SLS, was designed to measure the degree that one finds satisfaction in their life, as opposed to the SHS, which measures reflections of one’s purported feelings of happiness. Five items are rated on a 7-point Likert scale ranging from “1 = strongly disagree” to “7 = strongly agree”. Higher scores indicate higher levels of life satisfaction. Research verifies the measure to have good internal consistency and test–retest reliability in adolescents (Diener et al., 1985).

*Self-Compassion Scale for Youth*. Neff et al.’s (2021) SCSY measures self-compassion through multiple factors representing both positive and negative domains. Three factors—mindfulness, common humanity, and self-kindness—contribute to a positive subscale of self-compassion, while another the three factors—overidentification, isolation, and self-judgement—contribute to a negative subscale that is antithetical to self-compassion. After reverse scoring the negative factor responses, all six factors contribute to a total, global score. The measure utilizes 17 items to capture these factors, which are each ranked on a scale of “1 = almost never” to “5 = almost always”, with higher scores indicating greater levels of self-compassion. Fan et al. (2022) showed the measure to have good internal consistency with adolescents.

*Coping Through Emotional Approach Scale*. Stanton et al.’s (2000) CTEAS is a 54-item measure of coping strategies designed as factors that represent attunement with emotions, such as processing, expressing, and seeking social support. Higher scores on the subscales represent greater levels of emotional coping. Two specific factors, emotional processing and emotional expression, were used in this study to represent coping abilities that should theoretically coincide with PF, comprising 8 items with a Likert-type scale that ranged from “1 = I usually don’t do this at all” to “4 = I usually do this a lot”. These CTEAS scales demonstrate good internal consistency reliability in adolescents (Stanton et al., 2000).

*Difficulties in Emotion Regulation Scale—18*. Victor and Klonsky’s (2016) DERS-18 is an 18-item form of emotion regulation skills, including awareness, clarity, goals, impulse, nonacceptance, and strategies. The DERS uses a Likert-type scale ranging from “1 = almost never” to “5 = almost always”, and higher scores indicate greater levels of emotional dysregulation. The current study utilized a 9-item version of the measures that included lack of emotional awareness, clarity, and acceptance to represent difficulties with emotional regulation that correlates with PI (Barr, 2022). Initial research demonstrates good internal consistency reliability (Victor & Klonsky, 2016).

#### 2.1.4. Data Analysis

The first phase of data analysis was conducting an EFA on the 60 MPFI items. A pattern matrix of standardized factor loadings was analyzed, and eigenvalues were evaluated through parallel analyses and scree tests. An oblique rotation was utilized due to its allowance of freely estimated structures with potentially correlated factors (Flora & Flake, 2017). Cross-loading items were removed, factor loading coefficients lower than 0.30 were removed, and items with the strongest variable coefficients across each factor were retained, all to identify the most parsimonious measurement model (Flora & Flake, 2017). Other forced structures were conducted to evaluate the goodness of fit for other possible factor representations. The final model was determined through evaluation of empirical evidence in alignment with present theory (Kline, 2014). After an optimal measurement model was identified, descriptive statistics and bivariate correlations of new subscales were calculated.

The second phase of data analysis examined the convergent and divergent validity of the new model structure with measures of related constructs. Zero-order Pearson’s r correlations were calculated between subscale scores (derived from the EFA results) and scores from these other measures. All analyses were conducted in Jamovi version 2.5, which is a free, open statistical interface utilizing R programming language (The Jamovi Project, 2024).

### 2.2. Results

#### 2.2.1. Preliminary Analyses

Observations for this study were used in previous research, so datasheets were previously cleaned, and summed composite scores were calculated. No missing data or outliers were identified. Descriptive statistics verified that EFA and Pearson’s r assumptions were met (see Table 1). Skewness and kurtosis estimates for each measure were considered normal if ≤|3.0| (Kline, 2014), and internal consistency omega estimates ≥ 0.70 were deemed acceptable. No measure was noted as inappropriate for use in these present analyses, and primary analyses were executed as planned.

#### 2.2.2. Exploration of Latent Structure

*Unconstrained EFA.* A latent factor structure utilizing the original 60 MPFI items was tested via a series of EFA iterations using a maximum likelihood extraction method in combination with a Promax rotation. The results from an initial unconstrained analysis utilizing parallel analysis showed strong Kaiser–Meyer–Olkin (KMO) sampling adequacy (0.92), and Bartlett’s test of sphericity indicated no concerning issues of normality (*p* < 0.001).

An initial, unconstrained EFA presented a five-factor solution explaining 54.5% of the total variance. Thirty total items, representing present-moment awareness, self-as-context, defusion, values, committed action, and experiential avoidance, loaded onto a first factor. Sixteen total items, representing self-as-content, fusion, lack of contact with values, inaction, and acceptance, loaded onto a second factor. Some 12 items, representing acceptance, present-moment awareness, self-as-context, defusion, and lack of present-moment awareness, loaded onto a third factor and cross-loaded with the first and second factors. All items representing lack of present-moment awareness uniquely loaded onto a fourth factor, and all items representing self-as-content loaded onto a fifth factor, cross-loading with the second factor.

Inspection of eigenvalues and a visual scree plot for these initial results indicated a notably strong, two-factor solution of positive flexibility (inversely including experiential avoidance in the structure) and negative inflexibility (inversely including acceptance in the structure).

*Constrained EFAs.* Additional constrained EFAs followed to test the possibility of other existing factor structures. Forced iterations started with 12 factors, as modeled in the traditional MPFI, to highlight groups of items theorized to represent the total 12 processes of PF and PI. Results for the forced 12 factors did not match theory as the solution suggested an isolated acceptance factor, an isolated lack of present-moment awareness factor, and broad flexibility and inflexibility factors sharing multiple cross-loadings with items across scattered, semi-structured process domains. Most notably, eigenvalues suggested the strong presence of a two-factor, general PF and PI structure.

A forced six-factor solution was then conducted to test the presence of a structure represented by open, aware, engaged, non-open, non-aware, and non-engaged factors (Levin et al., 2014). This forced solution did not match theory as the solution presented a strong positive flexibility factor that included all items of present-moment awareness, defusion, self-as-context, values, committed action, and experiential avoidance, as well as a strong negative flexibility factor that included some cross-loaded acceptance items, self-as-content, lack of contact with values, and inaction. A clear factor representing lack-of-present-moment awareness and acceptance was highlighted as a third factor, and a few items representing self-as-content and fusion were highlighted as a fourth factor but cross-loaded significantly with general flexibility and inflexibility. Again, eigenvalues suggested the strong presence of a two-factor structure, and the forced six-factor solution failed to maintain a comprehensive open/aware/engaged and non-open/non-aware/non-engaged structure.

After that, a forced two-factor solution was conducted to evaluate the presence of a broad, dual-structure representation of PF and PI. Loadings for this constrained model showed all items from present-moment awareness, defusion, self-as-context, values, committed action, and experiential avoidance loading on the first factor, which mostly represented PF. Items from lack of present-moment awareness, fusion, self-as-content, lack of contact with values, inaction, and acceptance loaded onto a second factor, which mostly represented PI.

Finally, a forced 1-factor solution elicited a general PI factor in which acceptance item 1 and all items from lack-of-present-moment awareness, fusion, self-as-content, lack-of-contact with values, and inaction loaded together. No other items loaded above the 0.30 threshold.

After testing all theoretically coherent solutions, forced models including three, four, five, seven, eight, nine, ten, and eleven factors were additionally examined for other conceptual possibilities. None of these additional models yielded theoretical coherence. However, each model consistently maintained the presence of a strong two-factor structure representing PF and PI. These results together confidently suggest that a two-factor model is most viable for these data.

*EFA Model Refinement.* After selecting the two-factor model as preferred, additional modifications were made to refine the model, balancing empirical and theoretical considerations. When testing different solutions, a notable pattern presented in which all items related to acceptance loaded on either their own factor, loaded on the PI factor, or demonstrated insufficient loadings. Similarly, experiential avoidance items loaded consistently with the PF factor. This unusual finding alluded to results reported by Barr (2022), in which experiential avoidance scores correlated positively with other scales of PF and wellbeing, and acceptance scores correlated positively with other scales of PI and psychopathology. Given these findings, the experiential avoidance and acceptance items were removed from the EFA model due to being empirically problematic to the overarching measurement structure and interpretation of latent variables.

All other processes related to PF and PI were empirically represented in this two-factor structure. A constrained two-factor solution with all remaining 50 items showed strong sampling adequacy (KMO = 0.93) and explained 50% of the cumulative variance. Interestingly, the interfactor correlation was calculated near zero (ϕ = −0.005).

The item content was further refined to test if removal of weaker functioning or conceptually redundant items would lead to substantive changes in the underlying structure. Items with cross-loadings, weaker loadings, and redundant content were removed in a simultaneous fashion, which for the PF factors included removing present-moment awareness items 2, 3, and 5; self-as-context items 3, 4, and 5; defusion items 1, 4, and 5; values items 2, 4, and 5; and committed action items 2, 3, and 4. Items with cross-loadings, weaker loadings, and redundant content were removed in a similar, simultaneous fashion for the PI factor, which included removing lack of present-moment awareness items 2, 3, 4, and 5; self-as-content items 3, 4, and 5; fusion items 2, 3, and 4; lack of contact with values items 1, 3, and 4; and inaction items 1 and 5.

The final EFA model revealed two factors indicated by the 20 strongest and most unique items from the original 60-item MPFI, with 10 items representing PF and 10 representing PI (see Table 2). The final EFA model demonstrated adequate sampling adequacy (KMO = 0.89), explained 49.2% of the cumulative variance, and similarly showed a near-zero interfactor correlation (ϕ = 0.053). The PF factor included 2 items each from the original present-moment awareness, defusion, self-as-context, values, and committed action scales of the MPFI. The PI factor included 2 items each from the original fusion, self-as-content, and lack of contact with values scales, along with 1 item from the lack of present-moment awareness scale and 3 items from the inaction scale. The standardized factor loadings for this final 20-item model are presented in Table 2.

The descriptive statistics for these newly observed scores verified adequate sampling characteristics, including a normal distribution across the population, robust internal consistency reliability coefficients, and adequate factor/sum score correlations (see Table 3).

#### 2.2.3. Convergent and Divergent Validity

Zero-order bivariate Pearson’s *r* correlations between the 10-item PF scale with positive measures of wellbeing and emotion regulation (i.e., SHS, SLS, SCSY, and CTEAS) indicated consistent moderate to strong, positive correlations (see Table 4). Correlations between the 10-item PI scale with negative measures of internalizing psychopathology and emotion dysregulation (i.e., AAQ-II, AFQY, RCADS-25, and DERS) indicated consistent strong, positive correlations (see Table 4). Correlations between wellbeing measures and the PI scale were near zero or negative and small. Correlations between internalized psychopathology, emotion dysregulation, and the PF scale were near zero or negative and moderate (see Table 4).

### 2.3. Discussion

The purpose of Study 1 was to probe whether all processes of PF and PI contributed to broad constructs of PF and PI in a near-nationally representative sample of adolescents with diverse racial/ethnic identities. The results demonstrate that a two-factor structure of PF and PI is most empirically robust and theoretically sound (see Table 2). In the preferred 20-item measurement model, 2–3 items each have been shown to represent present-moment awareness, defusion, self-as-context, values, and committed action to contribute to a broad factor representing PF. Similarly, 2–3 items each have been shown to represent a lack of present-moment awareness, fusion, self-as-content, contact with values, and inaction contribute to a broad factor representing PI. The descriptive statistics of scores derived from these scales yield normal distributions and strong internal consistency reliability (see Table 3). These observed score distributions are comparable to distributions from the general PF and PI factors found in Rolffs et al.’s (2018) MPFI that was administered to adolescents, which are originally reported in Barr (2022). PF scores consistently correlated with theoretically related measures of wellbeing and emotional regulation, and PI scores correlated with measures of psychopathology and emotional dysregulation (see Table 4). These present correlations that are comparable to those reported in Barr (2022) suggest that the brief measure’s scores converge and diverge similarly to the full-length measure’s scores.

These initial findings are notable in determining a simplified structure upon which PF and PI might be measured in adolescents, of which no work has yet provided evidence-based guidance (Petersen et al., 2023). To date, only Greco et al.’s (2008) AFQY has been structurally validated as a measure partially representing PI in adolescents, but the structure focuses on experiential avoidance and cognitive fusion rather than PF or PI. These findings show that items—linked to most processes—significantly contribute to overarching PF and PI constructs.

That said, processes representing acceptance—in connection to PF—and experiential avoidance—in connection to PI—did not demonstrate theoretical alignment with the present sample. These counter-intuitive findings align with Barr’s (2022) validation of the MPFI in older adolescents, in which acceptance scores correlated strongly and positively with psychopathology and dysregulation scores, whereas experiential avoidance scores correlated strongly and positively with wellbeing and regulation scores. Developmental research highlights adolescence as a unique time of mental health that is different from adults (L. L. Hayes & Ciarrochi, 2015). Perhaps the developmental contexts of teenagers elicit strategies of acceptance and experiential avoidance different from adults, or the item content could be too abstract for adolescent comprehension. Whatever the case, measurement of acceptance and experiential avoidance using the current MPFI may be inappropriate with youth. Study 2 of this current manuscript was important to replicate this finding in a larger, clinically diverse sample of adolescents since this finding may have implications for the effective, ethical practice of measuring and interpreting PF and PI with younger populations.

Finally, the interfactor correlation between PF and PI in the reduced 20-item measurement model was near zero. This interesting and counter-intuitive finding demonstrates, on the group level, that youth may simultaneously report high levels of flexibility and high levels of inflexibility or low levels of flexibility and high levels of inflexibility, with no ability to predict a pattern or relationship between these variables. This suggests that PF and PI might not be assumed as polar opposites of a single dimension, as also suggested in Ong and Eustis (2023). However, this finding also proposes these two factors are not related, which is different than being distinct yet inversely related.

It may be the case that a non-specific nature of the item content promotes reflections of times in which youth are both flexible and inflexible. For example, youth responding to each question might reflect on times when they were flexible while learning in school yet simultaneously reflect on times of being inflexible in social settings outside of school. It is often the case that individuals are not entirely flexible or inflexible across all moments of their life, and flexibility depends, in part, on the amount of avoidance they feel towards certain, specific stimuli (S. C. Hayes et al., 2006). If this near-zero correlation is found again in a different, larger sample of adolescents, research might examine context-specific or developmental appropriateness of the item wording as a potential moderator for such variability. For example, items might be more tailored towards specific youth contexts, such as “When I am at school, negative thoughts and feelings tended to stick with me for a long time” or “When I am by myself, I was attentive and aware of my emotions”.

Both the issue of acceptance and experiential avoidance processes failing to contribute to PF and PI factors and the near-zero correlation of summed scores required replication before considerations for practice and policy were drawn. These issues are re-examined in Study 2, in alignment with the initial goals, including the confirmation of a simplified factor structure, re-examining convergent and divergent validity, and addressing predictive validity.

## 3. Study 2

### 3.1. Materials and Methods

#### 3.1.1. Participants and Data Collection

Prior to the survey administration, the reading levels for all measures were tested using the online Readability Analyzer application (Tyler, n.d.). The readability results indicated reading abilities of at least middle-school level or around 13 years old. Therefore, the target sample was a large group of adolescents aged 14–17 years. The study’s sample of adolescents approximately represented gender, race, and location demographics across the United States, as indicated by the most recent census data (see Table 5). Age was evenly distributed across the sample, such that approximately 25% of the sample was from each year (see Table 5). Further demographic information, including reported counts of sexual identity, English as a native language, socio-economic status, and living status, are reported in Table 5.

Sample size recommendations allowing adequate power in CFA vary depending on the number of items being administered and one’s anticipated factor structure. A common rule is collecting 5–10 participants per measure item (Bentler & Chou, 1987), so analyzing the MPFI with 60 items would require a sample size of 300–600 participants. Based on preliminary findings from the correlated two-factor structure from Study 1, Wolf et al. (2013) recommend a sample size of approximately 450. These considerations, in accordance with available funding, resulted in a desired sample size of approximately 500 participants.

Approval was obtained from the university’s Institutional Review Board prior to obtaining data. The collection utilized national survey panels, organized in partnership by research teams from the online platform Qualtrics, which attempted to recruit as close to a nationally representative sample as possible in regard to racial/ethnic identity of the adolescents, based on estimates from Qualtrics that were based on the most recent United States census survey. Qualtrics advertised the survey via various electronic sources, such as targeted email lists, website intercept, gaming sites, etc. Guardians of adolescent participants were sent an email invitation or prompted on their respective online platform to proceed with a given survey and mention an incentive that is offered. Parental consent, followed by youth assent, was collected prior to study participation. Following data collection, subsequent cleaning and removal of inappropriate responses finalized a sample size of 503 participants.

The online survey presented youth with a series of rating scales measuring PF and PI, wellbeing, internalized psychopathology, and suicide risk, which are detailed in the Measures Section. Participants were asked to answer questions honestly and accurately according to their personal experiences, and data collection ended when participants answered all question items. The total survey administration took approximately 26 min to complete (*M* = 26.83, *SD* = 30.92), with 23 outliers taking longer than 60 min. The dataset was downloaded from Qualtrics panel distributors, and the data were cleaned and prepared for analyses.

#### 3.1.2. Measures

*Demographics*. Participant demographic information was collected at the beginning of survey administration, and questions were written in accordance with diversity considerations recommended by Hughes et al. (2022). Many of these demographic questions were also utilized in the regression series as covariates.

*Multidimensional Psychological Flexibility Inventory*. See the prior description of the MPFI provided in Study 1.

*Avoidance and Fusion Questionnaire for Youth—8*. See the prior description of the AFQY provided in Study 1.

*Self-Compassion Scale for Youth*. See the prior description of the SCSY provided in Study 1.

*Generalized Anxiety Disorder Screener–7*. The GAD-7 is a frequently used screener of generalized anxiety disorder in medical settings in adolescent populations (Spitzer et al., 2006). The screener contains seven items with a 4-point Likert-type scale of “0 = Not at all” to “3 = Nearly every day”, with higher scores equating to higher risk of anxiety disorder. Examples of items include “Over the past two weeks, how often have you been bothered by the following problems? Feeling nervous, anxious, or on edge”, “trouble relaxing”, and “being so restless it is hard to sit still”. The screener shows strong evidence for construct, factorial, and criterion validity, as well as good reliability (Spitzer et al., 2006), and is widely used and accepted as a gold standard screener for youth anxiety problems with good internal reliability estimates. Various readability analyses, including the Flesch–Kinaid Grade Level, Fry Readability Grade Level, and Dale–Chall Score, indicate that the GAD-7 requires approximately a middle-school reading level (aged approximately 12–13 years) to comprehend.

*Patient Health Questionnaire–9*. The PHQ-9 (Kroenke et al., 2001) is a widely utilized screener of depression severity in medical settings for adolescent populations. The PHQ-9 contains nine items that represent the nine criteria for major depressive disorder listed in the DSM-IV. The measure utilizes a 4-point Likert-type scale of “0 = Not at all” to “3 = nearly every day”, with higher scores equating to higher risk of major depressive disorder. Examples of items begin with the qualifier: “Over the last two weeks, how often have you been bothered by any of the following problems?” and item examples include “Little interest or pleasure in doing things”, “Feeling tired or having little energy”, and “Thoughts that you would be better off dead or of hurting yourself in some way”. Like the GAD-7, the PHQ-9 shows strong evidence for construct, factorial, and criterion validity, as well as good reliability (Kroenke et al., 2001). The measure is widely used and accepted to be a gold standard screener for youth depression struggles. Various readability analyses, including the Flesch–Kinaid Grade Level, Fry Readability Grade Level, and Dale–Chall Score, indicate that the PHQ-9 requires approximately an older-elementary reading level (aged approximately 11 years) to comprehend.

*Suicide Risk*. Indicators for suicide risk were taken from the Ask Suicide Screening Questions (ASQ; National Institute of Mental Health, 2024), representing current suicide risk and past suicide attempts. Question 1 asks, “Has there been a time in the past month when you have had serious thoughts about ending your life?” Question 2 asks, “Have you ever, in your whole life, tried to kill yourself or made a suicide attempt?” These questions are valid marks of suicide risk and commonly used in outpatient and primary care settings (Aguinaldo et al., 2021). Both questions are responded to by checking “Yes” or “No”, and these responses were used as binomial regression outcomes, representing current suicide risk and past suicide attempts.

*Brief Multidimensional Students’ Life Satisfaction Scale*. The BMSLSS (Huebner et al., 2006) is a six-item measure of life satisfaction across various domains of a youth’s life, including family, friendships, school, individual identity, living situation, and life broadly. Items assess each domain individually and utilize a 7-point Likert-type scale with “1 = Terrible” and “7 = delighted” and a total score indicating total level of life satisfaction across domains, with higher scores equating to higher life satisfaction. Examples of items include “I would describe my satisfaction with my family life as…” and “I would describe my satisfaction with my school experience as…” This measure is used widely across school and other youth mental health settings to assess and screen for youth wellness, and research provides evidence of internal consistency and factorial structure, as well as convergent validity with other measures of youth wellness (Zullig et al., 2001; Seligson et al., 2003). Various readability analyses, including the Flesch–Kinaid Grade Level, Fry Readability Grade Level, and Dale–Chall Score, indicate that the BMSLSS requires approximately a high school reading level (aged approximately 14 years) to comprehend.

*Positive and Negative Affect Schedule—Child Form*. The PANAS-C (Laurent et al., 1999) is a traditional measure of contrasting positive and negative affect in youth. The measure includes individual items stating different facets of mood, such as interested, afraid, excited, ashamed, upset, happy, and strong, that are divided across two individual factors: positive affect (PANAS+) and negative affect (PANAS−). The rate at which individuals experienced the emotions in the past week are rated with a 5-point Likert-type scale ranging from “1 = Not much or not at all” to “5 = A lot”, with higher scores equating to higher levels of positive affect for one domain and higher levels of negative affect in the other domain. The measure has shown evidence for good validity and reliability across multiple populations and has been validated in youth populations (Laurent et al., 1999; Wilson et al., 2014). Various readability analyses, including the Flesch–Kinaid Grade Level, Fry Readability Grade Level, and Dale–Chall Score, indicate that the PANAS requires approximately a middle-school reading level (aged approximately 13+ years or older) to comprehend.

#### 3.1.3. Data Analysis

*Preliminary Analyses*. After cleaning and organizing the data, preliminary analyses were conducted, including descriptive statistics, internal consistency reliability, Kendall’s tau-b correlations between measure items, and Pearson’s *r* correlations between summed composite scores.

*Examination of Structural Validity*. After confirming all statistical assumptions, the first phase of analysis consisted of conducting two CFA on responses to the MPFI: one evaluating the two-factor, correlated 20-item model and the other testing the original 12-factor, 60-item higher-order model. Given the categorical nature of responses to the MPFI, CFA were fit using the Weighted Least Squares estimator with latent variance adjustments (WLSMV; Mîndrilâ, 2010). According to traditional guidelines from Hu and Bentler (1999), a model’s global goodness of fit is determined using decision rules for multiple fit indices, including an RMSEA less than 0.06, an SRMR less than 0.08, a CFI greater than 0.90, and a TLI greater than 0.90. Items were determined as representative of their latent factors based on standardized loadings greater than 0.70 being ideal and 0.40–0.70 being acceptable (Kline, 2014). The fit indices for the shortened MPFI-Y and traditional MPFI models were compared to evaluate the relative structural validity of one model over another.

*Examination of Convergent and Divergent Validity*. The second phase of data analysis investigated evidence for convergent and divergent validity of observed scores from the 20-item MPFI-Y structure and the traditional 60-item MPFI in relation to each other and with other measures of mental health problems and wellbeing by conducting a series of Pearson’s r correlations. Guidelines for interpreting the strength of Pearson’s r correlation coefficients were taken from traditional recommendations for the social sciences: 0.00–0.19 represents a weak correlation, 0.20–0.49 represents a moderate correlation, and 0.50–0.99 represents a strong correlation.

*Examination of Predictive Validity*. The third and final phase of analysis was running six multiple linear and binomial regression models to investigate the usefulness of the observed scores from the 20-item MPFI-Y’s measurement model predicting clinically meaningful variables: wellbeing, measured by the BMSLSS (life satisfaction) and the PANAS+ (positive affect); internalized psychopathology, measured by the GAD-7 (anxiety) and the PHQ-9 (depression); and suicide risk, represented by the two suicide questions asking (1) current suicide risk and (2) past suicide attempts. The demographic variables mentioned above (age, gender, LGBTQ+ status, race, English as a second language, SES, location, and living status) have been documented as significant variables predicting youth mental health severity (Rothenberg et al., 2023; Weist et al., 2000; Weeks et al., 2020). Each variable that maintained adequate statistical power was used as a covariate to control for predictors of mental health in these regression series.

Each regression model was conducted with a 3-step procedure, in which Step 1 evaluated the overall model and individual characteristics of demographic covariates predicting outcome variables. After controlling for these demographic covariates at Step 1, comparisons were conducted across all models to test the informational value provided by the MPFI-Y PF scale (Step 2) and MPFI-Y PI scale (Step 3), each predicting every clinically relevant variable (wellbeing, internalizing psychopathology, and suicide risk).

Each linear and binomial regression was evaluated at the total model and individual predictor levels. For each linear regression, *R*^2^ was evaluated at the model level to understand the estimated variation explained in each outcome by the set of covariates and predictors at each step. F-tests evaluated the statistical significance of every step within each model. Other data–model fit indices were calculated to evaluate relative and global fit of model steps, including the Akaike information criteria (AIC), Bayesian information criteria (BIC), and root mean square error (RMSE). Binomial regressions do not produce *R*^2^ coefficients, so χ^2^-tests were used instead to evaluate overall statistical significance for every step in each model. Global data-fit indices were also calculated to observe the relative fit of each model step, including McFadden’s pseudo-*R*^2^ (*R*_McF_^2^), AIC, BIC, and deviance.

At the predictor level, for linear regression, unstandardized (b) and standardized (β) estimates were evaluated to understand how unit changes in predictors explained continuous variation in life satisfaction, positive affect, anxiety, and depression, with t-tests offering additional information about statistical significance of each covariate and predictors. For binomial regression, odds ratios (OR) were evaluated to understand how unit changes in predictors contributed to changes in the odds of endorsing “Yes” vs. “No” to experiencing current suicide ideation and past suicide attempts, whereas Z-tests further verified the statistical significance of each covariate and predictor. For all regression models, the results were considered (at both model and predictor levels) from Step 1 to Step 2 (comparing demographic covariates to PF) and then Step 2 to Step 3 (comparing PF to PI).

#### 3.1.4. Data Analysis Tools

The descriptive statistics, correlations, and regression models were conducted in Jamovi version 2.4, which is a free, open statistical interface built on top of traditional R programming language (The Jamovi Project, 2024). Jamovi was incapable of conducting latent confirmatory analyses using the necessary estimator for the observed data, so CFAs were conducted using the Lavan package (v0. 6–7; Rosseel, 2012) within the R working environment version 4.2 (R Core Team, 2022).

### 3.2. Results

#### 3.2.1. Preliminary Analyses

The initial descriptive statistics suggest that all continuous variables (i.e., observed scores of PF, PI, self-compassion, and mental health outcomes) were adequately normally distributed, and most scales presented excellent internal consistency reliability, with a few presenting good or adequate reliability (see Table 6). The sample size was large enough, and the categorical variables (suicide risk and past suicide attempts) were sufficiently represented to allow the use of binomial regression (Pituch & Stevens, 2015).

Regarding demographic variables as covariates for the regression series, non-native English speakers consisted of 3% of the sample (see Table 5); therefore, this proportion did not maintain adequate power for the study, so the English as a second language variable was removed as a covariate. In a similar interest for maintaining adequate statistical power, those who answered “Unsure” to being LGBTQ+ status were combined with those who answered “Yes”. Likewise, those who identified as Asian, American Indian, Pacific Islander, Mixed, and prefer not to answer each were underpowered in regression analyses (see Table 5), so all were combined as an “other” race category. Finally, the Transgender variable was represented by four participants, so these responses were removed from the sample, bringing the size to N = 499 for these regression series. In summary, age (14, 15, 16, and 17), gender (male and female), LGBTQ+ status (no and yes + unsure), race (White, Black, Latinx, and Other), SES (Yes and No), location (Midwest, Northeast, South, and West), and living status (large neighborhood, big city, and small town) were utilized as covariates for Step 1 in the regression series.

Kendall’s tau-b correlation coefficients between all MPFI-Y items indicated theoretically consistent relationships across all PF indicators (items 1–10), correlating in the positive direction and with moderate to strong magnitude (range = 0.32–0.59). All PI indicators (items 11–20) likewise correlated in the positive direction, with moderate to strong magnitude (range = 0.31–0.76). The flexibility and inflexibility relationships generally ranged around a zero correlation, with some showing a theoretically consistent negative direction and weak relationship (range = −0.24–0.14).

Pearson’s *r* bivariate correlation coefficients between the associated measures of inflexibility and self-compassion indicated a strong relationship in a theoretically consistent direction (*r* = −0.53). The measures of self-compassion, life satisfaction, happiness, and positive affect correlated in the positive direction with strong, significant magnitudes (range = 0.45–0.69, *p*s < 0.001). The measures of inflexibility, depression, anxiety, and negative affect correlated in the positive direction with strong, significant magnitudes (range = 0.71–0.83, *p*s < 0.001). The relationships between wellbeing and psychopathology further demonstrated negative directionality with weak to strong, significant magnitudes (range = −0.12–−0.56, *p*s < 0.001).

#### 3.2.2. Structural Validity

**Figure 1:**
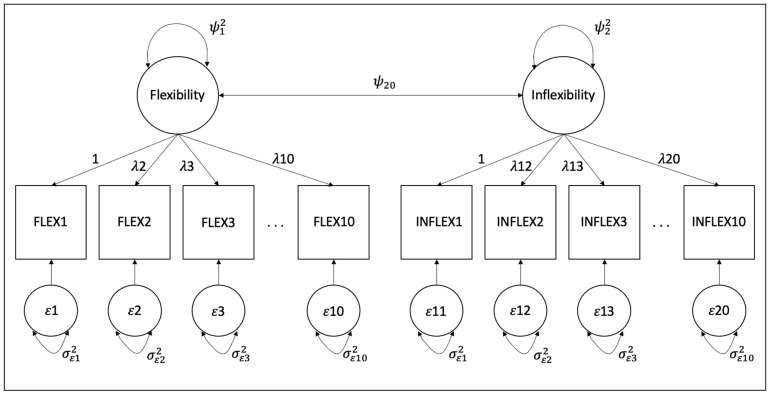
Simplified MPFI-Y two-factor measurement model. *Note*. MPFI-Y = Multidimensional Psychological Flexibility Inventory for Youth; FLEX = flexibility; INFLEX = inflexibility.

*The 20-Item MPFI-Y Model*. A correlated 20-item, two-factor MPFI-Y measurement model (see Figure 1), with items loading on respective latent variables of PF (Flexibility; 10 items) and PI (Inflexibility; 10 items), was conducted using categorical CFA with the WLSMV estimator. The results presented a strong global model fit (CFI = 0.984; TLI = 0.983; RMSEA [90% CI] = 0.045 [0.038, 0.052]; SRMR = 0.064), an adequate to strong standardized factor loadings with minimal range (Flexibility λ = 0.611–0.779; Inflexibility λ = 0.511–0.877; see Table 7), and a near-zero interfactor correlation (ϕ = −0.036).

The modification indices suggested that the model’s χ^2^ would decrease by 67 through the loading of Item 11 (representing lack of present-moment awareness) with the flexibility latent construct. The investigation of this item’s bivariate relationships with all other measure items showed reasonable discrimination of the item, correlating positive and moderately with other items of inflexibility, whereas positive yet zero to weak with items representing flexibility. This demonstration of correlative discrimination and an already adequate model fit characteristics did not warrant sufficient justification for loading the lack of present-moment awareness in this non-theoretical manner. Likewise, the modification indices recommended that Item 3 (representing self-as-content) be loaded with PF, but a similar decision was concluded. Similar patterns were observed for the modification indices recommending Item 9 (representing committed action) and Item 4 (representing self-as-context) as loading with the inflexibility construct. This notable modification index score may suggest evidence of model misspecification, but the investigation of item bivariate correlations otherwise shows theoretical consistency in the model as is. These correlational relationships between MPFI-Y items, taken with the adequate global fit indices and desire to maintain parsimony in the measurement model, suggest that the 20-item model could be maintained without making additional changes suggested by the modification index.

**Figure 2:**
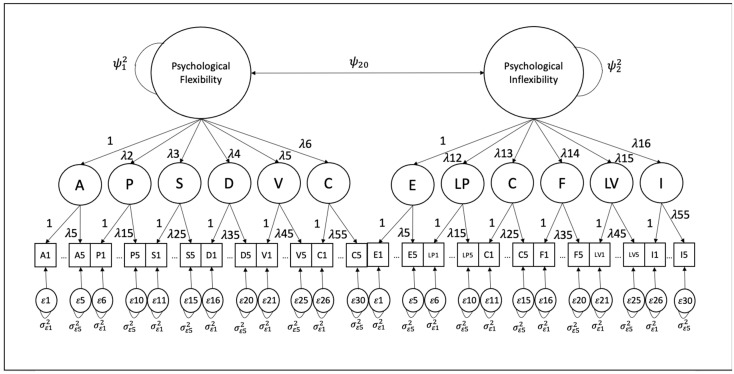
Traditional MPFI 12-factor, higher-order measurement model. *Note*. MPFI = Multidimensional Psychological Flexibility Inventory, A = acceptance, P = present-moment awareness, S = self-as-context, D = defusion, V = values, C = committed action, E = experiential avoidance, LP = lack of present-moment awareness, C = self-as-content, F = fusion, LV = lack of contact with values, I = inaction.

*The 60-Item MPFI Model.* A higher-order 60-item, traditional MPFI measurement model was additionally analyzed. This model loaded five items each onto 12 first-order factors representing two higher-order, correlated PF and PI processes (see Figure 2). The results from a similar CFA with the WLSMV estimator showed poor data–model fit (CFI = 0.915; TLI = 0.911; RMSEA [90% CI] = 0.097 [0.095, 0.098]; SRMR = 0.117).

Standardized item loadings ranged widely from weak to strong across the acceptance factor (0.396–0.852) but had a more minimal range and were consistently strong across all other factors representing processes of PF and PI (0.710–0.924). For the higher-order processes, standardized item loadings ranged from 0.542–0.955 for first-order latent processes predicting PF and 0.268–0.933 for first-order latent processes predicting PI. The higher-order factors of PF and PI shared a near-zero interfactor correlation (ϕ = 0.061). The modification indices suggested that the model’s χ^2^ would be significantly reduced by over 6000 through loading the experiential avoidance factor with PF instead of PI and the acceptance factor with PI instead of PF. Other modification indices recommended regressing the experiential avoidance factor with other PF factors and regressing the acceptance factor with other PI factors.

#### 3.2.3. Convergent and Divergent Validity

Pearson’s *r* correlation coefficients between the MPFI-Y PF scale and positive-related measures of wellness, including self-compassion, life satisfaction, happiness, and positive affect, were all in the positive direction and between moderate to strong magnitudes (range = 0.45–0.54, *p*s < 0.01; see Table 8). The correlations between the traditional MPFI PF composite scale and positive-related measures of wellbeing, likewise, were in the positive direction and between moderate to strong in magnitude (range = 0.41–0.53, *p*s < 0.001). The correlations between the MPFI-Y PF scale and measures of wellbeing were consistently slightly stronger than the same relationships with the MPFI PF composite scale (Δ range = 0.01–0.04; see Table 8).

The correlations between the MPFI-Y PF scale and measures of inflexibility, negative affect, depression, and anxiety were all in the negative direction, ranging weak to moderate in magnitude (range = −0.9–−0.23, *p*s < 0.05). The correlations between the traditional MPFI PF composite scale and measures of psychopathology all trended in the negative direction but ranged at near zero to weak magnitudes (range = −0.03–−0.16; see Table 8), with varying levels of statistical significance. The correlations between the MPFI-Y PF composite scale and negative measures of psychopathology were consistently slightly stronger than the same relationships with the traditional MPFI PF composite scale (Δ range = 0.06–0.07; see Table 8).

The correlations between the MPFI-Y PI scale and measures of wellbeing were all in the negative direction, ranging weak to strong in magnitude (range = −0.12–−0.51, *p*s < 0.01; see Table 8). The correlations between the MPFI inflexibility composite scale and measures of wellbeing were likewise in the negative direction but ranging from near-zero to moderate in magnitude (range = −0.05–−0.45), with one relationship not significant but the other three significant at *p* < 0.001 (see Table 8). The correlations between the MPFI-Y PI summed score and measures of wellbeing were the same or consistently slightly stronger than the same relationships with the MPFI PI composite scale (Δ range = 0.00–0.08; see Table 8).

The correlations between the MPFI-Y PI summed score and measures of psychopathology were all in the positive direction and strong in magnitude (range = 0.65–0.72, *p*s < 0.001; see Table 8). The correlations between the MPFI PI composite score and measures of psychopathology were likewise in the positive direction and strong in magnitude (range = 0.60–0.69, *p* < 0.001; see Table 8). The correlations between the MPFI-Y PI summed score and measures of psychopathology were consistently slightly stronger than the same relationships with the MPFI PI composite score (Δ range = 0.03–0.05; see Table 8).

The MPFI-Y PF summed score correlated strongly and positively with the traditional MPFI PF composite sum (*r* = 0.97, *p* < 0.001). Likewise, the MPFI-Y PI summed score correlated strongly and positively with the traditional MPFI PI composite sum (*r* = 0.97, *p* < 0.001). The MPFI-Y PF summed score correlated positively and weakly with the traditional MPFI PI composite sum (*r* = 0.10, *p* < 0.05). The MPFI-Y PI summed score correlated positively with near zero magnitude and non-significantly with the traditional MPFI PF composite sum (*r* = 0.05, *p* > 0.05). The MPFI-Y PF summed score correlated with the MPFI-Y PI score non-significantly in the negative direction with near zero magnitude (*r* = −0.02, *p* > 0.05). The traditional MPFI PF composite sum correlated with the traditional MPFI PI composite sum significantly in the positive direction with weak magnitude (*r* = 0.16, *p* < 0.001).

#### 3.2.4. Predictive Validity

*Model-Level Results.* Every linear regression model indicated that each hierarchal step in model building was statistically significant in explaining substantial proportions of variance across all mental health outcomes (*p*s < 0.001; see Table 9). The set of demographic covariates at Step 1 explained a good proportion of variation for each mental health outcome (*R*^2^ = 0.097–0.122). Adding MPFI-Y PF in Step 2 resulted in meaningful, large improvements in variation explained for life satisfaction and positive affect (*R*^2^ = 0.250, 0.249) and meaningful, small improvements in variation explained for anxiety and depression (*R*^2^ = 0.048, 0.036; see Table 9). Adding MPFI-Y PI in Step 3 resulted in meaningful, small improvements in variation explaining life satisfaction and positive affect (R2 = 0.077, 0.016) and meaningful, large improvements in variation explaining anxiety and depression (*R*^2^ = 0.346, 0.333; see Table 9). The variation across life satisfaction and positive affect explained by MPFI-Y PF surpassed that explained by demographic covariates and MPFI-Y PI. The variance across anxiety and depression explained by MPFI-Y PI surpassed that explained by individual and MPFI-Y PF. All data–model fit indices showed that increasing steps generally resulted in better fitting models, and statistical value was added and confirmed across all model comparisons (results available via request to the authors).

The model level results for the binomial regression series showed similar trends. For both current suicide risk and past suicide attempts, Steps 1–3 were statistically significant, with data-fit indices showing improved fit at each step and χ^2^ increasing most drastically at Step 3 (see Table 10). The model comparison results for binomial regressions specified Step 3 as offering significant value to explaining both outcomes, but Step 2 failed at offering statistically significant value (results available via request to the authors). Binomial regressions do not permit determinations regarding variation explained for each outcome. That said, patterns in model fit results suggest that MPFI-Y PI (but not MPFI-Y PF) had meaningful associations with the current risk of suicide and past attempts of suicide (results available via request to the authors).

*Predictor-Level Results: Linear Regression Series.* The predictor level results for the linear regression models are presented in Table 11 (life satisfaction), Table 12 (positive affect), Table 13 (anxiety), and Table 14 (depression). In light of the multi-step building process, these tables only show results from the final model (Step 3) of each series. A step-by-step narrative summary of key findings from earlier models (Step 1 and Step 2) is presented to inform greater understanding of the final models (Step 3) presented in the aforementioned tables.

At Step 1, a few demographic covariates showed statistically significant associations with levels of life satisfaction, positive affect, anxiety, and depression. Reporting “Yes” or “I’m not sure” to identifying as LGBTQ+ was statistically significant in predicting lower levels of life satisfaction (*p* < 0.001; β = −0.76), lower levels of positive affect (*p* < 0.001; β = −0.55), greater levels of anxiety (*p* < 0.001; β = 0.78), and greater levels of depression (*p* < 0.001; β = 0.79). Identifying as mixed race resulted in statistically decreased levels of life satisfaction compared to White race (*p* = 0.041; β = −0.29), and living in a rural environment resulted in significantly decreased levels of life satisfaction compared to a suburban environment (*p* = 0.048; β = −0.23). Identifying as mixed race resulted in statistically decreased levels of positive affect compared to White race (*p* = 0.009, β = −0.36) and living in a rural area likewise resulted in decreased positive affect compared to a suburban area (*p* = 0.009; β = −0.29). Interestingly, living in an urban area compared to a suburban area was related to increased levels of positive affect (*p* = 0.013; β = 0.23). Reporting “Yes” to receiving free/reduced priced lunch resulted in increased levels of both anxiety (*p* < 0.001; β = 0.32) and depression (*p* = 0.002; β = 0.29). All other variables across outcomes (i.e., gender, other races aside from mixed race, and location) were not statistically significant across models at Step 1.

At Step 2, higher levels of MPFI-Y PF significantly predicted all outcome variables including increased life satisfaction (*p* < 0.001; β = 0.51), increased positive affect (*p* < 0.001; 0.51), decreased anxiety (*p* < 0.001; β = −0.22), and decreased depression (*p* < 0.001; β = −0.19). Reporting “Yes” or “Unsure” to identifying as LGBTQ+ maintained statistical significance for both anxiety (*p* < 0.001; β = 0.75) and depression (*p* < 0.001; β = 0.76) but attenuated slightly for both life satisfaction (*p* < 0.001; β = −0.68) and positive affect (*p* < 0.001; β = −0.47). Increased age emerged as a slight, significant predictor of decreased life satisfaction (*p* = 0.046; β = −0.08) and positive affect (*p* = 0.021; β = −0.08), and rural living maintained significance in predicting decreased positive affect (*p* = 0.008; β = −0.26). Reporting “Yes” to free/reduced priced lunch additionally maintained statistical significance for prediction of both increased anxiety (*p* < 0.001; β = 0.33) and increased depression (*p* < 0.001; β = 0.31), whereas urban living status significantly predicted increased depression (*p* = 0.013; β = 0.27).

At Step 3, MPFI-Y PF remained as a meaningful predictor across all outcome variables: life satisfaction (*p* < 0.001; β = 0.50), positive affect (*p* < 0.001; β = 0.50), anxiety (*p* < 0.001; β = −0.19), and depression (*p* < 0.001; β = −0.16; see Table 11, Table 12, Table 13 and Table 14). MPFI-Y PI added value for predicting decreased life satisfaction (*p* < 0.001; β = −0.30) and decreased positive affect (*p* < 0.001; β = −0.14) and greatly increased predictive value for increased anxiety (*p* < 0.001; β = 0.63) and increased depression (*p* < 0.001; β = 0.62; see Table 11, Table 12, Table 13 and Table 14). Reporting “Yes” or “Unsure” to identifying as LGBTQ+ maintained statistical significance but decreased in strength across all four outcomes (see Table 11, Table 12, Table 13 and Table 14). Increased age held significance in predicting both decreased life satisfaction and decreased positive affect (see Table 11, Table 12, Table 13 and Table 14), and rural living predicted both decreased life satisfaction and decreased positive affect (see Table 11, Table 12, Table 13 and Table 14). Identifying as “other race” (i.e., American Indian, Asian, Polynesian, or prefer not to answer) became an additional, statistically significant predictor of decreased positive affect as compared to identifying as “White” (see Table 12), and identifying as female emerged as a statistically significant predictor of increased anxiety compared to identifying as male (see Table 13).

Predictor-Level Results: Binomial Regression Series. The predictor level results for the binomial regression series are presented in Table 15 (current suicide risk) and Table 16 (past suicide attempts). Similar to the linear regression series, these tables only present results from the final model (Step 3) of each outcome. A step-by-step narrative summary of key findings from the earlier models (Step 1 and Step 2) is presented below to provide greater context for understanding the results from the final models (Step 3).

At Step 1, LGBTQ+ status was a statistically significant predictor of current suicide risk (*p* = 0.009; OR = 2.26) and past suicide attempts (*p* = 0.002; OR = 2.90), indicating that youth reporting “Yes” or “Not Sure” to identifying as LGBTQ+ had about two to three times the odds of experiencing current suicide risk or past suicide attempts compared to youth reporting “No”.

Likewise, identifying as Black was a statistically significant predictor of both current suicide risk (*p* = 0.036; OR = 0.3) and past suicide attempts (*p* = 0.048; OR = 0.23), indicating approximately 70–80% increased odds of experiencing current suicide risk or past suicide attempts compared to youth identifying as White. No other demographic covariates presented as statistically significant in predicting current ideation or past attempts.

At Step 2, MPFI-Y PF alone did not present as a statistically significant predictor of current suicide risk (*p* = 0.076; OR = 0.98) or past suicide attempts (*p* = 0.129; OR = 0.978). LGBTQ+ status remained as a statistically significant predictor of both current suicide risk (*p* = 0.012; OR = 2.20) and past suicide attempts (*p* = 0.003; OR = 2.81). Identifying as Black also remained as a predictor of both current suicide risk (*p* = 0.035; OR = 0.37) and past suicide attempts (*p* = 0.047; OR = 0.22). No other demographic covariates changed their predictive potential in notable ways or presented as statistically significant in predicting current suicide ideation or past suicide attempts.

At Step 3, MPFI-Y PF became a statistically significant predictor of both current suicide risk (*p* = 0.020; OR = 0.97) and past suicide attempts (*p* < 0.025; OR = 0.97), indicating every 1-point increase in a PF score was associated with approximately 3% decreased odds in selecting “Yes” for experiencing both current ideation and past attempts (see Table 15 and Table 16). MPFI-Y PI presented as a statistically significant predictor of both current risk (*p* < 0.001; OR = 1.08) and past attempts (*p* < 0.001; OR = 1.09), indicating every 1-point increase in a PI score was associated with approximately 8–9% increased odds in selecting “Yes” for experiencing both current ideation and past attempts (see Table 15 and Table 16). LGBTQ+ status became non-significant in predicting both current suicide ideation (*p* = 0.286; OR = 1.44; see Table 15) and past suicide attempts (*p* = 0.131; OR = 1.79; see Table 16). Identifying as Black also became a non-significant predictor of current risk (*p* = 0.057; OR = 0.39; see Table 15) but maintained a significant predictor of past attempts (*p* = 0.045; OR = 0.20; see Table 16).

### 3.3. Discussion

The purpose of Study 2 was to replicate findings from Study 1 and confirm that the simplified representation of PF and PI in adolescents maintained a robust structure, convergent and divergent validity, and useful predictive validity. As expected, the CFA results confirm that the MPFI-Y maintains a strong factor representation of PF and PI. On the other hand, the traditional MPFI 12-factor structure presented a relatively poor model fit. The modification indices especially highlighted the problematic nature of acceptance loading with PF and experiential avoidance loading with PI, significantly recommending model improvement through experiential avoidance instead loading with PF and acceptance with PI. This unusual finding was first identified in Barr (2022), where the observed scores representing acceptance and experiential avoidance correlated non-theoretically, suggesting that acceptance was more likely related to PI and psychopathology and experiential avoidance was more likely related to PF and wellbeing. Then, this finding was found again in results from Study 1, when items representing acceptance were loaded with both PF and PI factor constructs and items representing experiential avoidance were loaded with PF. Thus, this third identification of this issue in a separate, larger study confirms that problematic relationships likely exist in the measurement of acceptance and experiential avoidance processes in adolescents.

This surprising yet consistent finding suggests that the field of psychology might reconsider definitions and behavioral criterions of acceptance and experiential avoidance, as measured by the MPFI in adolescent populations. No research prior to this study has modeled or verified the measurement of acceptance as predicting global levels of PF and PI in children and adolescents to date (Petersen et al., 2023). According to the findings in this study and in Barr (2022), current understandings of acceptance and experiential avoidance may be different in adults than the ways adolescents conceptualize them in self-report rating forms. On the other hand, many studies show that experiential avoidance and fusion measured by the AFQY are consistently predictive of many youth mental health struggles (e.g., Reddy et al., 2006), which maintains the theory that experiential avoidance may still be related to PI and youth psychopathology, assuming the construct is defined in a developmentally appropriate manner. Thus, problematic measurement of acceptance and experiential avoidance that is suggested by results in Barr (2022) and this present study may exist because of how the MPFI measures acceptance and experiential avoidance, which may be different from the how the AFQY measures experiential avoidance (e.g., employing different phrasing for item stems, item content, and response options).

Indeed, other measures of PF and PI have been shown to disagree in content (Ong et al., 2024). With such considerations in mind, a post hoc analysis of the current data was completed, in which the summed factor scores for acceptance and experiential avoidance were correlated with the AFQY total score. The results showed experiential avoidance had a positive, near-zero correlation with the AFQY score (r = 0.05), and acceptance had a positive, weak correlation with the AFQY score (r = 0.25). These findings suggest that higher ratings on items measuring acceptance in the traditional MPFI may be related to greater levels of experiential avoidance, as measured by the AFQY. The near-zero correlation shared between experiential avoidance on the MPFI and experiential avoidance defined on the AFQY shows that the two measures may, in fact, be measuring different constructs, as proposed by Ong et al. (2024).

Future studies examining comprehensive models of PF and PI in adolescents should test the correlation of other items that have been shown to represent acceptance and experiential avoidance with the current, simplified MPFI-Y structure. Future research should examine whether the inclusion of already developmentally adapted items representing experiential avoidance and acceptance items for adolescents prove to load in a more theoretically expected manner with the simplified structure of PF and PI than current items of acceptance and experiential avoidance in the traditional MPFI for adults.

The other items representing PF and PI in the simplified structure may be a good foundation to rely upon since these items maintained convergence with measures of theoretical similarity and divergence from measures of theoretical opposition. Particularly notable is the MPFI-Y’s PF summed score correlating as moderate to strong with all measures of wellbeing and positive affect but the same scale correlating in the negative direction with weak to moderate magnitude across all measures of psychopathology and negative affect. Likewise, the MPFI-Y’s PI summed score correlated positively and strongly with all measures of psychopathology and negative affect, and moderately to strongly and negatively with measures of wellbeing and positive affect. Such findings are promising in suggesting evidence of convergent and divergent validity for the PF and PI scale scores derived from the 20-item version of the MPFI with theoretically related mental health experiences of youth.

Most interesting, again, is the correlation between the MPFI-Y’s PF and PI scale scores being near zero, which replicates the findings from Study 1. Past conceptualizations have stated that PF and PI are related yet distinct, suggesting that they should not be considered constructs on polar opposites of a single dimension (Ong & Eustis, 2023) and should maintain a small to moderate negative correlation. The near-zero correlation found in this study alludes to the necessity of measuring PF and PI as separate scores in adolescents, as there is little signal or pattern in their relationship. Based on this replicated data, one cannot interpret a high score of PF to inversely indicate a low score in PI and vice versa. That said, the authors believe this measure may still be measuring PF and PI since correlations with related measures converged in theoretically meaningful ways. For example, PF correlated moderately to strongly and positively with measures of happiness, self-compassion, and emotion regulation, whereas PI correlated strongly and positively with other measures of experiential avoidance, psychopathology, and negative emotions.

As mentioned in Study 1, this near-zero correlation may be occurring because the youth responding to each question could be reflecting on times when they were flexible in one setting, yet simultaneously reflecting on times of being inflexible in a different setting, but future research should test this possibility. It is often the case that individuals are not entirely flexible or inflexible across all moments of their life, and flexibility depends, in part, on the amount of avoidance they feel towards certain, specific stimuli (S. C. Hayes et al., 2006). As an analogy, a recent study comparing the predictive power of subjective wellbeing defined broadly compared to subjective wellbeing defined at school showed that wellbeing defined at school was much more strongly predictive of desired academic outcomes than wellbeing broadly (Renshaw et al., 2024). Similarly, Weeks et al. (2025) recently demonstrated that adolescents’ perceptions of social climate at home substantially differed from perceptions of social climate at schools and that school climate was a much stronger predictor of life satisfaction compared with home climate. Taking a cue from these studies, future research might examine context-specific or developmental appropriateness of item wording as a potential moderator for the variability in the current and replicated near-zero correlation of PF and PI summed scores. For example, items might be more tailored towards specific youth contexts, such as “When I am at school, negative thoughts and feelings tended to stick with me for a long time” or “When I am by myself, I was attentive and aware of my emotions”. A stronger correlation between PF and PI might occur after focusing the item content on a specific context where PF and PI are related to certain clinical interests in particular populations (e.g., at school, at home, when alone, or with peers).

Findings from the set of regression analyses further suggest the MPFI-Y’s usefulness in predicting important youth mental health outcomes. The summed PF score contributed a significant portion of variance in predicting both youth life satisfaction and youth positive affect. Likewise, the summed PI score contributed a significant portion of variance to predicting both youth anxiety and youth depression. Decreased levels of PI explained less variance in predicting life satisfaction and positive affect, and decreased levels of PF explained less variance in predicting depression and anxiety. This is consistent with the previous literature showing various processes contributing to PF and PI as being related to similar youth outcomes (Ciarrochi et al., 2011). Research also shows PF and PI comprehensively predict adult wellbeing and psychopathological outcomes (Gloster et al., 2017; Ong & Eustis, 2023).

However, findings from these regression series further suggest that the clearest determination of measure interpretation is higher scores on the MPFI-Y’s PF factor and lower scores on the MPFI-Y’s inflexibility factor, predicting higher scores of life satisfaction and positive affect. Likewise, higher scores on MPFI-Y’s PI factor and lower scores on MPFI-Y’s PF factor are most effective at predicting higher levels of anxiety and depression. The findings additionally suggest that decreased levels of flexibility and increased levels of inflexibility together may prove slightly useful in predicting an endorsement of “Yes” for current suicide risk and/or past suicide attempts. Scores of PF and PI together were most predictive of clinical outcomes, and these findings allude again to the importance of measuring flexibility and inflexibility as separate, distinct constructs together and in directions associated with desired mental health targets (psychopathology vs. wellbeing) rather than assuming PF and PI as opposites ends of a single spectrum (Ong & Eustis, 2023).

#### Implications for Policy and Practice

Given all the evidence from Studies 1 and 2, the MPFI-Y may serve as a potentially effective tool for measuring PF and PI in adolescents, particularly for screening and progress monitoring purposes. This measure may not differentiate differences in processes as effectively as the traditional MPFI model, but evidence from this study indicates that low PF and high PI scores are predictive of greater youth internalizing psychopathology and suicide risk, and high levels of PF and low levels of PI are likely predictive of greater youth wellbeing. With further research to support norm-based or criterion-based cut scores, this measure could be implemented in educational and clinical settings to assist in identifying youth with greater psychopathological concerns who would benefit from interventions shown to bolster one’s PF and decrease PI. Such youth might be identified through universal or targeted screening within youth systems, such as schools and residential centers, or within service settings, such as primary care, outpatient therapy clinics, or medical emergency rooms. Relatedly, the MPFI-Y might be used as a form of progress monitoring to examine if changes in PF and PI occur in theoretically expected directions as a youth builds greater fluency in PF and decreases in PI. However, further research is needed to explore the sensitivity of these scores to change in response to intervention.

## 4. Conclusions

PF is a promising skill grounded on the principles of behavior change (S. C. Hayes et al., 2006). Reported levels of PF may be related to one’s ability to make behavioral pivots necessary for increasing one’s wellbeing (S. C. Hayes et al., 2006). Likewise, increases in PF’s inverse, PI, have been shown to relate to a host of psychopathologies (Levin et al., 2014). Screening for such a skill and its associated opposite cannot occur without first developing a comprehensive measure of PF and PI in youth since effective evaluations of psychological theory depend on the extent to which processes of interest can be comprehensively and efficiently measured (Connors et al., 2015; Youngstrom et al., 2017). The groundwork has been laid in measuring PF and PI as a self-reported trait in adults (Francis et al., 2016; Rolffs et al., 2018); however, related measurement and validation efforts of PF and PI in youth are sparse (Petersen et al., 2023). This manuscript reported on two studies that aimed to generate evidence for validating a measurement model of PF and PI in a general sample of older adolescents (Study 1) and then to confirm and extend the findings in a second, larger sample of adolescents with a greater diversity in age (Study 2).

In Study 1, EFA results showed a theory-consistent measurement model that was much simpler than the original 12-factor model of the MPFI and maintained representation of most PF and PI processes, besides acceptance and experiential avoidance. The successful 10-item factor representation of PF and 10-item factor representation of PI further maintained convergent and divergent relationships with related indicators of mental health. PF and PI, however, shared a near-zero correlation, suggesting the constructs to be completely distinct with zero pattern in relationship.

In Study 2, the measurement model derived from Study 1 was tested via CFA and showed good representation of the PF and PI factors. The observed scores derived from this model further converged and diverged in theoretically consistent directions, with associated indices of wellbeing and internalized psychopathology, and scores from the simplified scales proved useful in predicting desired youth mental health outcomes. The regression output clarified PF to be the strongest predictor of life satisfaction and positive affect, and PI to be the strongest predictor of anxiety, depression, current suicide risk, and past suicide attempts. Each factor added value to the prediction of all desired outcomes, but these findings recommend screening for PF with the perspective of predicting youth wellbeing and screening for PI with the purpose of predicting youth psychopathological concerns.

On the other hand, the traditional 60-item representation of PF and PI fostered a poor fit for conceptualizing these constructs with adolescents. The fit indices confirmed responses to the acceptance items loaded more representatively with PI, and responses to the experiential avoidance items loaded more representatively with PF. That said, both the MPFI-Y’s summed PF/PI score and traditional MPFI’s summed PF/PI composite shared a 0.97 correlation. This verified little divergence between the brief and comprehensive models. Similar to the first study, the MPFI-Y’s factor correlation between PF and PI was confirmed as near zero, suggesting the constructs to be virtually unrelated and not to be measured as polar opposites on a spectrum.

Taken together, these findings suggest that the MPFI-Y is likely a useful measure for screening and differentiating skill levels across a range of youth wellbeing experiences and psychopathological concerns. The brief nature allows for its administration across a range of school, residential treatment, primary care, and outpatient therapeutic settings. This measure may be useful in identifying youth who would benefit from interventions designated as effective in altering levels of PF and PI, such as those in Acceptance and Commitment Therapy. However, this endeavor should be approached with caution as much research is needed in validating these items with a broader range of youth with greater levels of clinical concerns and with youth of diverse cultural backgrounds. Further research is also needed to identify norm-based or criterion-based cut scores that could be used for screening purposes. The limitations in the current project also include the use of convenience sampling and the potential for self-report bias since adolescents were identified using targeted Qualtrics panels, and one cannot control for how adolescents choose to respond to rating forms. Future research would benefit from generalizing our approach to validity analyses with more diverse samples of adolescents in specific service settings, such as school districts, residential treatment centers, outpatient clinics, and primary care.

## Figures and Tables

**Table 1:** Descriptive statistics for all variables: Study 1.

Measure	Min, Max	*M*	*Mdn*	*SD*	Q1, Q3	Skew	Kurt	ω
MPFI Flexibility	36, 180	125	124	24.6	109, 145	−0.186	−0.255	0.952
MPFI Inflexibility	52, 167	103	101	28.5	81, 123	0.232	−0.756	0.955
AAQ-II	7, 49	24.3	24	10.2	16, 32	0.025	−0.997	0.922
AFQY	17, 82	49.8	51	14.2	39, 60	−0.153	−0.497	0.912
RCADS-25	25, 100	50.9	49	16.8	37, 63	0.442	−0.702	0.960
SHS	4, 28	18.9	19	4.59	16, 22	−0.361	0.477	0.778
SLS	5, 35	24.2	25	6.91	20, 29	−0.723	0.123	0.902
SCSY	19, 84	52.6	52	8.84	49, 56	0.009	2.34	0.828
CTEAS	8, 32	23	24	5.93	19, 28	−0.494	−0.390	0.902
DERS	9, 38	22.5	23	6.89	17, 28	−0.121	−0.716	0.767

*Note*. These descriptive statistics are taken from the summed total composite score for each measure. AAQ-II = Acceptance and Action Questionnaire, 2nd Edition; AFQY = Avoidance and Fusion Questionnaire for Youth, 17 Items; RCADS-25 = Revised Child Anxiety and Depression Screener—25; SHS = Subjective Happiness Scale; SLS = Satisfaction with Life Scale; SCSY = Self-Compassion Scale for Youth; CTEAS = Coping Through Emotional Approach Scale; DERS = Difficulties in Emotion Regulation Scale—18; Min, Max = minimum and maximum; *M* = mean; *Mdn* = median; *SD* = standard deviation; Q1 = first and third quartiles; Skew = skewness; Kurt = kurtosis; ω = McDonald’s omega internal consistency coefficient.

**Table 2:** EFA pattern matrix for the final reduced MPFI measurement model: Study 1.

Item	Factor (*λ*)	
	Inflex (*ξ*1)	Flex (*ξ*2)
I did most things on “automatic” with little awareness of what I was doing.	**0.508**	0.101
I thought some of my emotions were bad or inappropriate and I shouldn’t feel them.	**0.571**	0.133
I criticized myself for having irrational or inappropriate emotions.	**0.549**	0.036
Negative thoughts and feelings tended to stick with me for a long time.	**0.713**	−0.105
When something bad happened it was hard for me to stop thinking about it.	**0.688**	−0.058
When life got hectic, I often lost touch with the things I value.	**0.818**	−0.064
When times got tough, it was easy to forget about what I truly value.	**0.779**	0.039
Negative feelings easily stalled out my plans.	**0.814**	−0.019
Getting upset left me stuck and inactive.	**0.864**	0.073
Negative experiences derailed me from what’s really important.	**0.860**	−0.080
I was attentive and aware of my emotions.	0.002	**0.636**
I was in touch with the ebb and flow of my thoughts and feelings.	0.080	**0.641**
Even when I felt hurt or upset, I tried to maintain a broader perspective.	0.055	**0.719**
I carried myself through tough moments by seeing my life from a larger viewpoint.	0.035	**0.687**
When I was upset, I was able to let those negative feelings pass through me without clinging to them.	−0.006	**0.614**
When I was scared or afraid, I was able to gently experience those feelings, allowing them to pass.	−0.040	**0.686**
I was very in-touch with what is important to me and my life.	−0.070	**0.683**
I tried to connect with what is truly important to me on a daily basis.	−0.110	**0.629**
Even when I stumbled in my efforts, I didn’t quit working toward what is important.	0.010	**0.708**
I didn’t let my own fears and doubts get in the way of taking action toward my goals.	0.033	**0.669**
Eigenvalues	5.342	4.063
% Variance	26.6	22.6

*Note*. The maximum likelihood extraction method was used with a Promax rotation. Bold = loadings identified on primary factor.

**Table 3:** Descriptive statistics for the condensed model of flexibility and inflexibility: Study 1.

Statistic	Inflex Scale	Flex Scale
Minimum, Maximum	10, 58	13, 60
Mean	32.8	42.7
Median	32	43
Standard Deviation	11.2	8.84
Quartile 1, Quartile 3	23, 42	36, 50
Skewness	0.139	−0.146
Kurtosis	−0.839	−0.382
Shapiro–Wilk *p*	<0.001	0.028
McDonald’s ω	0.917	0.889
Factor/Sum Score *r*	0.986	0.998

*Note*. These descriptive statistics are taken from the summed total composite score for each factor of the condensed model of psychological flexibility. Flex = psychological flexibility scale; Inflex = psychological inflexibility scale; Skew = skewness; Kurt = kurtosis; Shapiro–Wilk = Shapiro–Wilk test of normality.

**Table 4:** Pearson’s *r* coefficients for MPFI-Y scales with criterion variables: Study 1.

Criterion	Flex Scale	(95% CI)	Inflex Scale	(95% CI)
AAQ-II	−0.143 *	(−0.263, −0.019)	0.756 **	(0.697, 0.805)
AFQY	0.093	(−0.032, 0.215)	0.703 **	(0.634, 0.760)
RCADS-25	−0.062	(−0.185, 0.063)	0.672 **	(0.598, 0.735)
SHS	0.472 **	(0.369, 0.563)	−0.261 **	(−0.373, −0.141)
SLS	0.471 **	(0.369, 0.563)	−0.047	(−0.170, 0.078)
SCSY	0.427 **	(0.320, 0.524)	−0.389 **	(−0.490, −0.279)
CTEAS	0.623 **	(0.541, 0.694)	0.009	(−0.115, 0.133)
DERS	−0.307 **	(−0.415, −0.190)	0.547 **	(0.453, 0.628)

*Note*. ** = significant at *p* < 0.001; * = significant at *p* < 0.05. Flex = psychological flexibility scale; Inflex = psychological inflexibility scale. AAQ-II = Acceptance and Action Questionnaire, 2nd Edition; AFQY = Avoidance and Fusion Questionnaire for Youth, 17 Items; RCADS-25 = Revised Child Anxiety and Depression Screener—25; SHS = Subjective Happiness Scale; SLS = Satisfaction with Life Scale; SCSY = Self-Compassion Scale for Youth; CTEAS = Coping Through Emotional Approach Scale; DERS = Difficulties in Emotion Regulation Scale—18.

**Table 5:** Participant demographic characteristics: Study 2.

Characteristic/Group	*N*	% of Total
*Age*		
14	119	23.7%
15	132	26.2%
16	122	24.3%
17	130	25.8%
*Reported Gender*		
Male	251	49.9%
Female	248	49.3%
Transgender	4	>1%
*Race*		
White American/European	238	47.3%
Hispanic/Latinx/Spanish	95	18.9%
Black/African American	54	10.7
Asian/Asian American	26	5.2%
American Indian	24	4.8%
Native Hawaiian/Pacific Islander	1	>1%
Mixed Race	63	12.5%
Prefer Not to Answer	2	>1%
*LGBTQ+ Identity*		
No	438	87.1%
Not Sure	7	1.4%
Yes	58	11.5%
*English at Home*		
Yes	484	96.2%
No	19	3.8%
*Reported Use of Free/Reduced Price Lunch*		
Yes	434	68.2%
No	160	31.8%
*Living Status*		
Urban	201	40.0%
Suburban	131	26.0%
Rural	171	34.0%
*Location*		
South	180	35.8%
Northeast	100	19.9%
Midwest	102	20.3%
West	121	24.1%

*Note*. *N* = 503 with no missing data.

**Table 6:** Descriptive statistics for concurrent outcome variables: Study 2.

Measure	Min, Max	*M*	*Mdn*	*SD*	Q1, Q3	Skew	Kurt	ω
MPFI-Y Flex	13, 60	40.9	41	9.67	34, 48	−0.147	−0.204	0.911
MPFI-Y Inflex	10, 60	28.7	27	11.8	20, 36	0.577	−0.181	0.940
TradMPFI Flex	40, 180	120	119	26.4	104, 137	−0.125	−0.049	0.963
TradMPFI Inflex	40, 180	92.2	88	30.9	70, 110	0.672	0.171	0.967
SCSY	20, 85	55.6	54	9.30	50, 61	0.349	1.090	0.827
BMSLSS	7, 42	30.9	32	6.24	27, 36	−0.761	0.830	0.889
SHS	4, 28	20	20	4.73	17, 23	−0.569	0.681	0.732
PANAS+	12, 60	42.4	44	10.8	35, 50	−0.395	−0.450	0.960
AFQY-8	0, 32	10.3	8	8.09	4, 15	0.815	−0.137	0.916
PANAS−	15, 75	29.4	24	13.8	18, 38	1.05	0.458	0.962
PHQ-9	0, 27	6.14	4	6.74	0, 10	1.07	0.364	0.946
GAD-7	0, 21	5.94	5	5.62	1, 9	0.873	0.026	0.943

*Note*. These descriptive statistics are taken from the summed total composite score for each measure. MPFI-Y Flex = Multidimensional Psychological Flexibility Inventory for Youth, flexibility factor; MPFI-Y Inflex = Multidimensional Psychological Flexibility Inventory for Youth, inflexibility factor; TradMPFI Flex = Traditional Multidimensional Psychological Flexibility Inventory, total flexibility composite; TradMPFI Inflex = Traditional Multidimensional Psychological Flexibility Inventory, total inflexibility composite; SCSY = Self-Compassion Scale for Youth; BMSLSS = Brief Multidimensional Student Life Satisfaction Scale; SHS = Subjective Happiness Scale; PANAS+ = Positive and Negative Affect Scale, Positive Affect Subscale; AFQY-8 = Avoidance and Fusion Questionnaire for Youth-8; PANAS− = Positive and Negative Affect Scale, Negative Affect Subscale; PHQ-9 = Patient Health Questionnaire-9; GAD-7 = Generalized Anxiety Disorder Screener-7. Min, Max = minimum and maximum; M = mean; Mdn = median; SD = standard deviation; Q1 = first and third quartiles; Skew = skewness; Kurt = kurtosis; ω = McDonald’s omega internal consistency coefficient.

**Table 7:** MPFI-Y two-factor CFA standardized loadings: Study 2.

Item	λ	*SE*	Var.
**Flexibility Factor**			
Q1	0.611	0.056	0.627
Q2	0.636	0.055	0.596
Q3	0.758	0.045	0.425
Q4	0.726	0.050	0.473
Q5	0.682	0.054	0.534
Q6	0.733	0.046	0.462
Q7	0.736	0.047	0.458
Q8	0.779	0.044	0.393
Q9	0.696	0.050	0.516
Q10	0.748	0.044	0.441
**Inflexibility Factor**			
Q11	0.511	0.065	0.739
Q12	0.668	0.063	0.554
Q13	0.761	0.053	0.420
Q14	0.750	0.054	0.437
Q15	0.796	0.049	0.366
Q16	0.870	0.044	0.243
Q17	0.826	0.047	0.318
Q18	0.877	0.046	0.230
Q19	0.857	0.048	0.266
Q20	0.847	0.047	0.283

*Note*. MPFI-Y = Multidimensional Psychological Flexibility Inventory for Youth. λ = standardized factor loading; *SE* = standard error of each standardized factor loading; Var. = variance of each factor loading.

**Table 8:** Pearson’s *r* coefficients for MPFI and MPFI-Y scales with mental health variables: Study 2.

	MPFI-Y Flex	MPFI Flex	*r*Δ Flex	MPFI-Y Inflex	MPFI Inflex	*r*Δ Inflex
SCSY	0.453 ***	0.432 ***	0.02	−0.511 ***	−0.454 ***	0.06
BMSLSS	0.520 ***	0.489 ***	0.03	−0.325 ***	−0.248 ***	0.08
SHS	0.451 ***	0.407 ***	0.04	−0.399 ***	−0.339 ***	0.00
PANAS+	0.547 ***	0.533 ***	0.01	−0.120 **	−0.047	0.07
AFQY-8	−0.091 *	−0.030	0.06	0.728 ***	0.699 ***	0.03
PHQ-9	−0.176 **	−0.106 *	0.07	0.650 ***	0.600 ***	0.05
GAD-7	−0.231 **	−0.161 ***	0.07	0.657 **	0.609 ***	0.05
PANAS−	−0.179 **	−0.109 *	0.07	0.691 **	0.654 ***	0.04

*Note*. *** = *p* < 0.001; ** = *p* < 0.01; * = *p* < 0.05. MPFI-Y = Multidimensional Psychological Flexibility Inventory for Youth (shortened version); MPFI = Multidimensional Psychological Flexibility Inventory (original version); Flex = flexibility scale; Inflex = inflexibility scale; SCSY = Self-Compassion Scale for Youth; BMSLSS = Brief Multidimensional Student Life Satisfaction Scale; SHS = Subjective Happiness Scale; PANAS+ = Positive and Negative Affect Scale, Positive Affect Subscale; AFQY-8 = Avoidance and Fusion Questionnaire for Youth–8; PANAS− = Positive and Negative Affect Scale, Negative Affect Subscale; PHQ-9 = Patient Health Questionnaire–9; GAD-7 = Generalized Anxiety Disorder Screener–7.

**Table 9:** Model level results for linear regression series.

Model/Step	*R* ^2^	AIC	BIC	RMSE	*F*	*df*_1_	*df*_2_	*p*
*Life Satisfaction*								
Step 1: Demographics	0.097	3247	3310	5.92	4.06	13	489	<0.001
Step 2: PF	0.348	3086	3153	5.04	18.57	14	488	<0.001
Step 3: PI	0.428	3024	3096	4.73	23.97	15	487	<0.001
*Positive Affect*								
Step 1: Demographics	0.121	3789	3852	10.15	5.19	13	489	<0.001
Step 2: PF	0.370	3623	3690	8.59	20.50	14	488	<0.001
Step 3: PI	0.387	3612	3683	8.48	20.48	15	487	<0.001
*Anxiety*								
Step 1: Demographics	0.111	3135	3198	5.30	4.69	13	489	<0.001
Step 2: PF	0.159	3109	3177	5.15	6.57	14	488	<0.001
Step 3: PI	0.505	2845	2916	3.96	33.07	15	487	<0.001
*Depression*								
Step 1: Demographics	0.122	3311	3374	6.31	5.21	13	489	<0.001
Step 2: PF	0.157	3292	3360	6.18	6.51	14	488	<0.001
Step 3: PI	0.490	3041	3113	4.81	31.20	15	487	<0.001

*Note*. AIC = Akaike information criteria, BIC = Bayesian information criteria, RMSE = root mean square error, PF = psychological flexibility, PI = psychological inflexibility.

**Table 10:** Model level results for binomial regression series.

Model/Comparison	$R_{McF}^{2}$	AIC	BIC	Deviance	*χ* ^2^	*df*	*p*
*Current Suicide Risk*							
Step 1: Demographics	0.049	518	577	490	25.1	13	0.023
Step 2: PF	0.055	517	580	487	28.2	14	0.013
Step 3: PI	0.161	464	532	432	83.2	15	<0.001
*Past Suicide Attempt*							
Step 1: Demographics	0.060	385	444	357	22.9	13	0.043
Step 2: PF	0.067	384	448	354	25.2	14	0.033
Step 3: PI	0.178	344	411	312	67.7	15	<0.001

*Note*. AIC = Akaike information criteria, BIC = Bayesian information criteria, PF = psychological flexibility, PI = psychological inflexibility.

**Table 11:** BMSLSS (life satisfaction): linear regression predictor results (Step 3).

Predictor	*b*	*SE*	*t*	*p*	*β*	95% CI_L_	95% CI_U_
Intercept	32.20	3.37	9.55	<0.001	—	—	—
Age	−0.57	0.20	−2.88	0.004	−0.10	−0.17	−0.03
Gender:							
Female–Male	0.115	0.45	0.25	0.799	0.02	−0.12	0.16
Sexual Identity							
Yes or Not Sure–No	−3.149	0.68	−4.64	<0.001	−0.51	−0.72	−0.29
Race:							
Black–White	0.183	0.73	0.25	0.803	0.03	−0.20	0.26
Hispanic–White	−0.248	0.60	−0.41	0.681	−0.04	−0.23	0.15
Other Race–White	−1.368	0.70	−1.96	0.051	−0.22	−0.44	0.01
Mixed Race–White	−0.193	0.76	−0.25	0.799	−0.03	−0.27	0.21
Free/Reduced Price Lunch							
Yes–Not	−0.025	0.48	−0.05	0.958	−0.01	−0.15	0.15
Location:							
Northeast–Midwest	0.574	0.69	0.84	0.402	0.09	−0.12	0.31
South–Midwest	−0.327	0.61	−0.54	0.593	−0.05	−0.25	0.14
West–Midwest	0.543	0.67	0.81	0.420	0.09	−0.13	0.30
Living Status:							
Urban–Suburban	−0.137	0.56	−0.24	0.807	−0.02	−0.20	0.16
Rural–Suburban	−1.229	0.57	−2.16	0.031	−0.20	−0.38	−0.02
MPFI-Y Flexibility	0.319	0.02	14.06	<0.001	0.50	0.43	0.56
MPFI-Y Inflexibility	−0.158	0.02	−8.09	<0.001	−0.30	−0.37	−0.23

*Note*. BMSLSS = Brief Multidimensional Student Life Satisfaction Scale, MPFI-Y = Multidimensional Psychological Flexibility Inventory for Youth, *SE* = standard error, CI_L_ = lower bound for confidence interval, CI_U_ = upper bound for confidence interval.

**Table 12:** PANAS+ (positive affect): linear regression predictor results (Step 3).

Predictor	*b*	*SE*	*t*	*p*	*β*	95% CI_L_	95% CI_U_
Intercept	39.208	6.04	6.49	<0.001	—	—	—
Age	−0.951	0.35	−2.68	0.008	−0.10	−0.17	−0.03
Gender:							
Female–Male	−1.156	0.81	−1.43	0.154	−0.11	−0.25	0.04
Sexual Identity							
Yes or Not Sure–No	−4.177	1.22	−3.43	<0.001	−0.39	−0.61	−0.17
Race:							
Black–White	0.211	1.32	0.16	0.873	0.02	−0.22	0.26
Hispanic–White	−1.114	1.08	−1.03	0.302	−0.10	−0.30	0.09
Other Race–White	−2.942	1.25	−2.35	0.019	−0.27	−0.50	−0.05
Mixed Race–White	−0.161	1.36	−0.12	0.906	−0.02	−0.26	0.23
Free/Reduced Price Lunch							
Yes–Not	1.293	0.85	1.51	0.130	−0.04	−0.04	0.28
Location:							
Northeast–Midwest	−0.997	1.23	−0.81	0.417	−0.09	−0.32	0.13
South–Midwest	−0.738	1.10	−0.67	0.501	−0.07	−0.27	0.13
West–Midwest	0.979	1.21	0.81	0.417	0.09	−0.13	0.31
Living Status:							
Urban–Suburban	1.267	1.01	0.21	0.210	0.12	−0.07	0.30
Rural–Suburban	−2.812	1.02	0.01	0.006	−0.26	−0.45	−0.08
MPFI-Y Flexibility	0.563	0.04	13.83	<0.001	0.50	0.43	0.57
MPFI-Y Inflexibility	−0.126	0.04	−3.61	<0.001	−0.14	−0.21	−0.06

*Note*. PANAS+ = Positive and Negative Affect Scale, Positive Affect Subscale, MPFI-Y = Multidimensional Psychological Flexibility Inventory for Youth, *SE* = standard error, CI_L_ = lower bound for confidence interval, CI_U_ = upper bound for confidence interval.

**Table 13:** GAD-7 (anxiety): linear regression predictor results (Step 3).

Predictor	*b*	*SE*	*t*	*p*	*β*	95% CI_L_	95% CI_U_
Intercept	−2.701	2.81	−0.96	0.339	—	—	—
Age	0.176	0.17	1.07	0.287	0.04	−0.03	0.01
Gender:							
Female–Male	0.902	0.38	2.39	0.017	0.16	0.03	0.29
Sexual Identity							
Yes or Not Sure–No	2.125	0.57	3.74	<0.001	0.38	0.18	0.58
Race:							
Black–White	0.198	0.61	0.32	0.748	0.04	−0.18	0.25
Hispanic–White	0.318	0.50	0.63	0.528	0.06	−0.12	0.23
Other Race–White	0.496	0.58	0.85	0.396	0.09	−0.12	0.29
Mixed Race–White	−0.116	0.64	−0.18	0.856	−0.02	−0.24	0.20
Free/Reduced Price Lunch							
Yes–Not	0.772	0.40	1.94	0.053	0.14	−0.01	0.28
Location:							
Northeast–Midwest	0.423	0.57	0.74	0.461	0.08	−0.13	0.28
South–Midwest	0.239	0.51	0.47	0.641	0.04	−0.14	0.22
West–Midwest	0.296	0.56	0.53	0.599	0.05	−0.14	0.25
Living Status:							
Urban–Suburban	0.082	0.47	0.17	0.863	0.02	−0.15	0.18
Rural–Suburban	0.270	0.48	0.57	0.571	0.05	−0.12	0.21
MPFI-Y Flexibility	−0.110	0.02	−5.78	<0.001	−0.19	−0.25	−0.13
MPFI-Y Inflexibility	0.301	0.02	18.44	<0.001	0.63	0.56	0.70

*Note*. GAD-7 = Generalized Anxiety Disorder–7 screener, MPFI-Y = Multidimensional Psychological Flexibility Inventory for Adolescents, *SE* = standard error, CI_L_ = lower bound for confidence interval, CI_U_ = upper bound for confidence interval.

**Table 14:** PHQ-9 (depression): linear regression predictor results (Step 3).

Predictor	*b*	*SE*	*t*	*p*	*β*	95% CI_L_	95% CI_U_
Intercept	−7.30	3.43	−2.13	0.034	—	—	—
Age	0.37	0.20	1.82	0.069	0.06	−0.01	0.13
Gender:							
Female–Male	0.87	0.46	1.90	0.058	0.13	−0.01	0.26
Sexual Identity							
Yes or Not Sure–No	2.67	0.69	3.86	<0.001	0.40	0.19	0.60
Race:							
Black–White	−0.72	0.75	−0.96	0.336	−0.11	−0.32	0.11
Hispanic–White	0.98	0.61	1.60	0.110	0.15	−0.03	0.32
Other Race–White	0.48	0.71	0.68	0.497	0.07	−0.14	0.28
Mixed Race–White	−0.08	0.77	−0.10	0.919	−0.01	−0.24	0.21
Free/Reduced Price Lunch							
Yes–Not	0.76	0.48	1.57	0.118	0.11	−0.03	0.25
Location:							
Northeast–Midwest	0.50	0.70	0.71	0.477	0.07	−0.13	0.28
South–Midwest	0.25	0.62	0.41	0.683	0.04	−0.14	0.22
West–Midwest	0.75	0.68	1.10	0.271	0.11	−0.09	0.31
Living Status:							
Urban–Suburban	0.71	0.57	1.23	0.218	0.11	−0.06	0.27
Rural–Suburban	0.04	0.58	0.08	0.939	0.01	−0.16	0.18
MPFI-Y Flexibility	−0.11	0.02	−4.80	<0.001	−0.16	−0.22	−0.09
MPFI-Y Inflexibility	0.35	0.02	17.82	<0.001	0.62	0.55	0.69

*Note*. PHQ-9 = Patient Health Questionnaire–9, MPFI-Y = Multidimensional Psychological Flexibility Inventory for Adolescents, *SE* = standard error, CI_L_ = lower bound for confidence interval, CI_U_ = upper bound for confidence interval.

**Table 15:** Current suicide risk: binomial regression predictor results (Step 3).

Predictor	Est.	*SE*	*Z*	*p*	OR	95% CI_L_	95% CI_U_
Intercept	−4.26	1.93	−2.21	0.027	0.01	<0.001	0.62
Age	0.10	0.11	0.84	0.400	1.10	0.88	1.37
Gender:							
Female–Male	0.14	0.26	0.55	0.586	1.15	0.70	1.90
Sexual Identity							
Yes or Not Sure–No	0.36	0.34	1.07	0.286	1.44	0.74	2.79
Race:							
Black–White	−0.95	0.50	−1.91	0.057	0.39	0.15	1.03
Hispanic–White	−0.31	0.34	−0.91	0.362	0.73	0.38	1.43
Other Race–White	−0.34	0.38	−0.89	0.373	0.71	0.34	1.51
Mixed Race–White	−0.68	0.47	−1.44	0.149	0.51	0.20	1.28
Free/Reduced Price Lunch							
Yes–No	0.19	0.28	0.68	0.498	1.21	0.70	2.09
Location:							
Northeast–Midwest	0.10	0.39	0.26	0.796	1.11	0.51	2.39
South–Midwest	0.12	0.35	0.30	0.766	1.11	0.56	2.22
West–Midwest	0.58	0.37	1.54	0.123	1.78	0.86	3.69
Living Status:							
Urban–Suburban	0.05	0.31	0.14	0.886	1.05	0.57	1.93
Rural–Suburban	−0.25	0.33	−0.75	0.452	0.78	0.41	1.50
MPFI-Y Flexibility	−0.03	0.01	−2.34	0.020	0.97	0.95	0.99
MPFI-Y Inflexibility	0.08	0.01	6.86	<0.001	1.08	1.06	1.11

*Note*. MPFI-Y = Multidimensional Psychological Flexibility Inventory for Adolescents, Est. = estimate of log odds, *SE* = standard error, OR = odds ratio, CI_L_ = lower bound for confidence interval, CI_U_ = upper bound for confidence interval.

**Table 16:** Past suicide attempts: binomial regression predictor results (Step 3).

Predictor	Est.	*SE*	*Z*	*p*	OR	95% CI_L_	95% CI_U_
Intercept	−4.40	2.35	−1.88	0.061	0.01	<0.001	1.22
Age	0.07	0.14	0.54	0.592	1.08	0.82	1.41
Gender:							
Female–Male	−0.06	0.31	−0.19	0.849	0.94	0.51	1.74
Sexual Identity							
Yes or Not Sure–No	0.58	0.38	1.51	0.131	1.79	0.84	3.80
Race:							
Black–White	−1.59	0.79	−2.01	0.045	0.20	0.04	0.96
Hispanic–White	−0.26	0.43	−0.60	0.552	0.77	0.33	1.80
Other Race–White	−0.22	0.46	−0.47	0.636	0.80	0.33	1.99
Mixed Race–White	−0.02	0.52	−0.04	0.972	0.98	0.36	2.70
Free/Reduced Price Lunch							
Yes–Not	0.27	0.35	0.77	0.440	1.31	0.66	2.61
Location:							
Northeast–Midwest	0.15	0.45	0.35	0.729	1.17	0.49	2.79
South–Midwest	−0.24	0.43	−0.57	0.567	0.78	0.34	1.80
West–Midwest	0.07	0.44	0.17	0.868	1.08	0.46	2.53
Living Status:							
Urban–Suburban	−0.35	0.38	−0.92	0.359	0.70	0.33	1.49
Rural–Suburban	−0.27	0.39	−0.68	0.497	0.77	0.35	1.65
MPFI-Y Flexibility	−0.04	0.02	−2.24	0.025	0.97	0.94	0.99
MPFI-Y Inflexibility	0.09	0.02	5.99	<0.001	1.09	1.06	1.12

*Note*. MPFI-Y = Multidimensional Psychological Flexibility Inventory for Youth, Est. = estimate of log odds, *SE* = standard error, OR = odds ratio, CI_L_ = lower bound for confidence interval, CI_U_ = upper bound for confidence interval.

## Data Availability

The raw data supporting the conclusions of this article will be made available by the authors on request.
